# Supplementary material for: Adaptation of the infant gut microbiome during the complementary feeding transition
Source: PLoS One. 2022 Jul 14;17(7):e0270213. doi: 10.1371/journal.pone.0270213 (PMC9282554; doi:10.1371/journal.pone.0270213)

## Slide 1
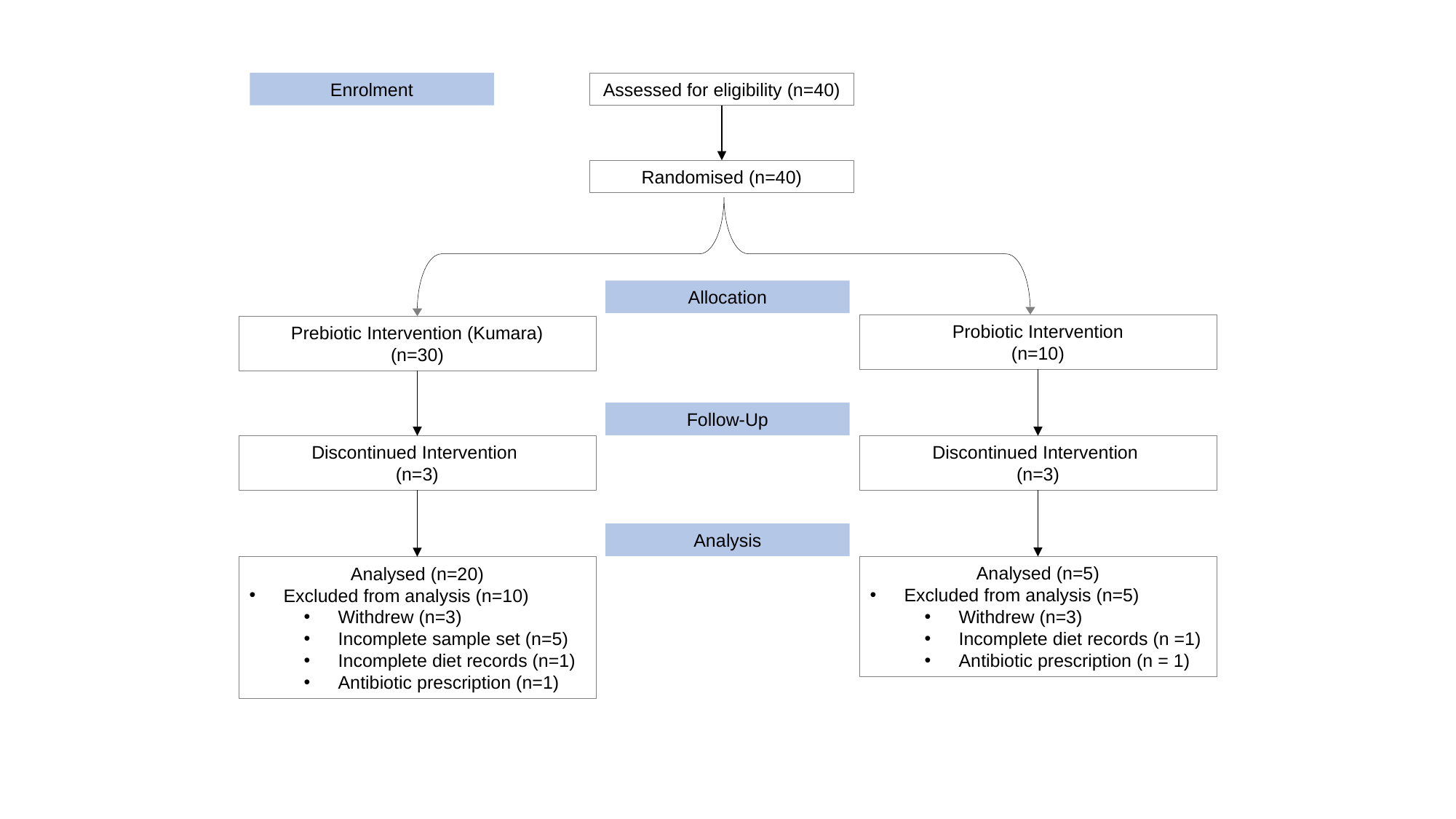

Enrolment
Assessed for eligibility (n=40)
Randomised (n=40)
Allocation
Probiotic Intervention
(n=10)
Prebiotic Intervention (Kumara)
(n=30)
Follow-Up
Discontinued Intervention
(n=3)
Discontinued Intervention
(n=3)
Analysis
Analysed (n=5)
Excluded from analysis (n=5)
Withdrew (n=3)
Incomplete diet records (n =1)
Antibiotic prescription (n = 1)
Analysed (n=20)
Excluded from analysis (n=10)
Withdrew (n=3)
Incomplete sample set (n=5)
Incomplete diet records (n=1)
Antibiotic prescription (n=1)

## Slide 2
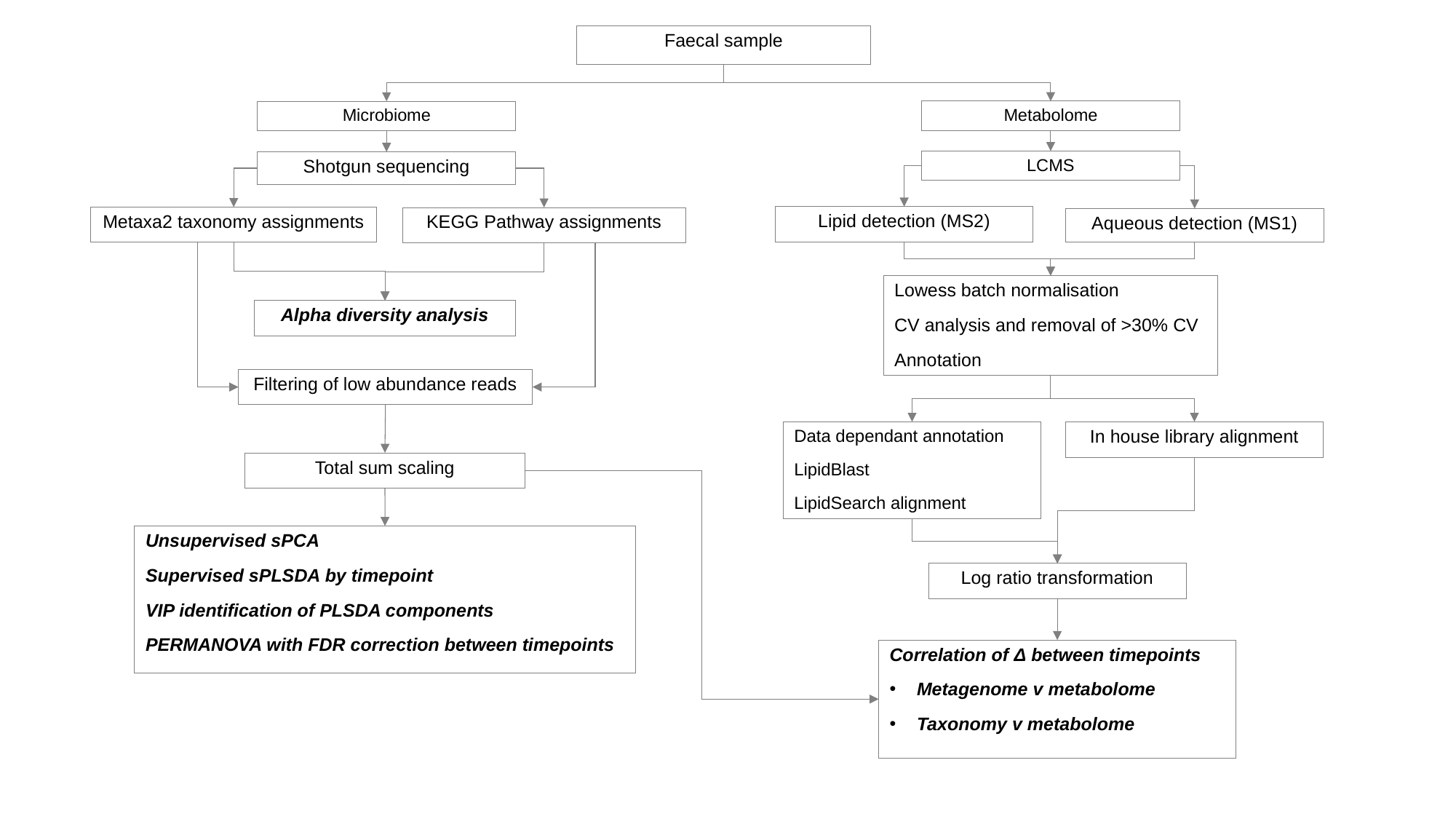

Faecal sample
Metabolome
Microbiome
LCMS
Shotgun sequencing
Lipid detection (MS2)
Metaxa2 taxonomy assignments
KEGG Pathway assignments
Aqueous detection (MS1)
Lowess batch normalisation
CV analysis and removal of >30% CV
Annotation
Alpha diversity analysis
Filtering of low abundance reads
Data dependant annotation
LipidBlast
LipidSearch alignment
In house library alignment
Total sum scaling
Unsupervised sPCA
Supervised sPLSDA by timepoint
VIP identification of PLSDA components
PERMANOVA with FDR correction between timepoints
Log ratio transformation
Correlation of Δ between timepoints
Metagenome v metabolome
Taxonomy v metabolome

## Slide 3
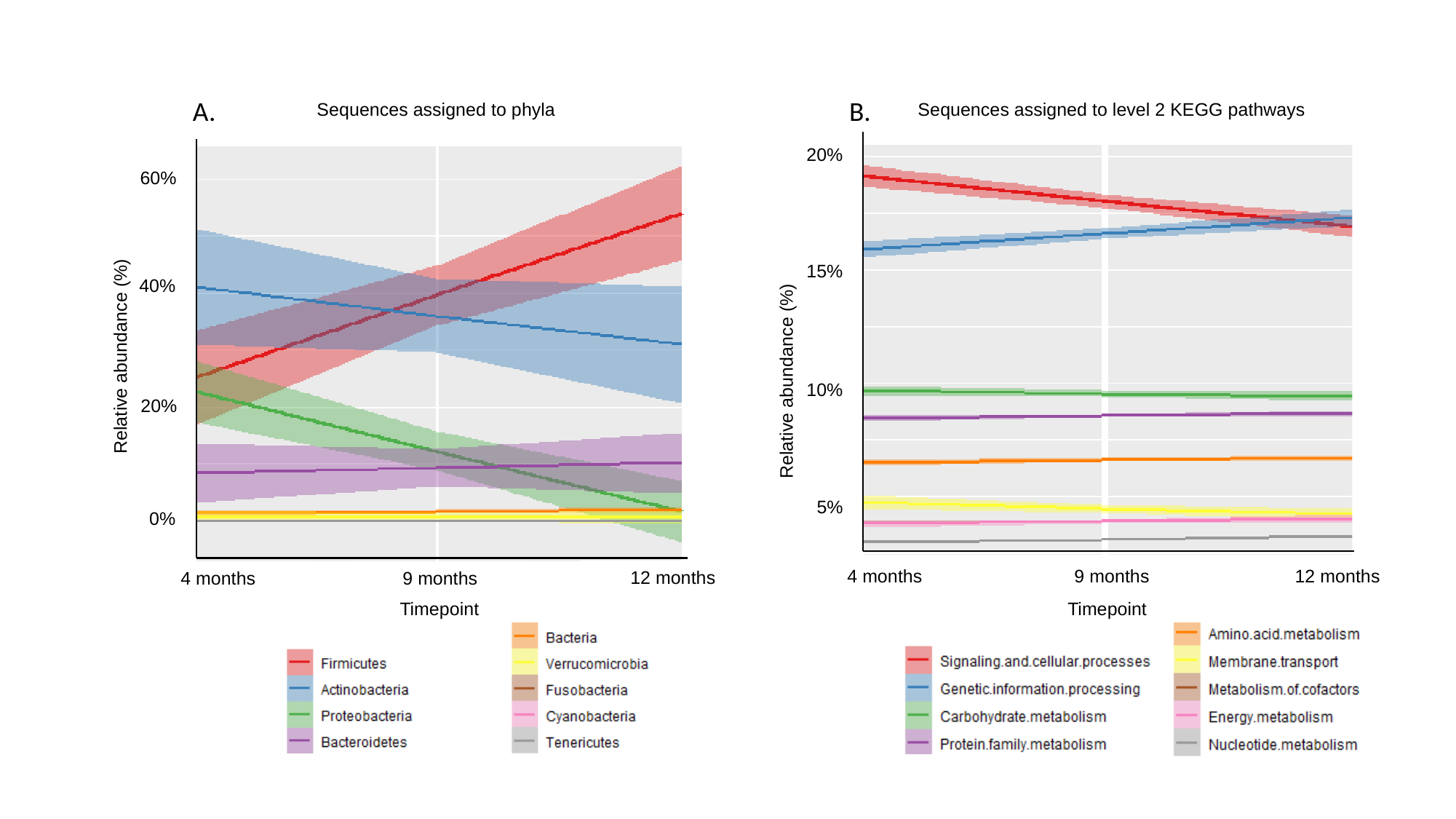

60%
Relative abundance (%)
40%
20%
0%
12 months
4 months
9 months
Timepoint
A.
B.
Sequences assigned to phyla
Sequences assigned to level 2 KEGG pathways
20%
Relative abundance (%)
15%
Relative abundance (%)
10%
5%
4 months
12 months
9 months
Timepoint

## Slide 4
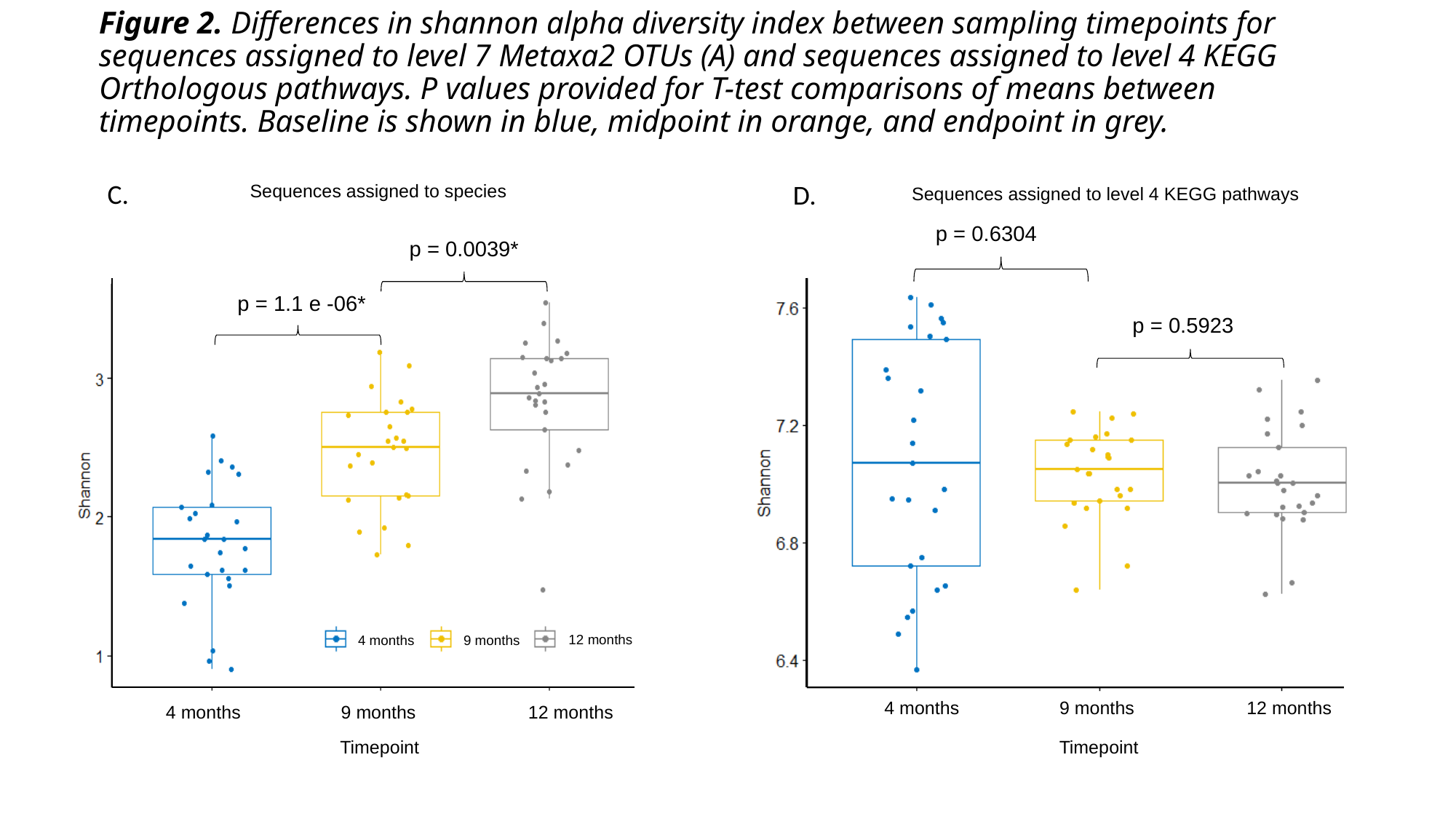

Figure 2. Differences in shannon alpha diversity index between sampling timepoints for sequences assigned to level 7 Metaxa2 OTUs (A) and sequences assigned to level 4 KEGG Orthologous pathways. P values provided for T-test comparisons of means between timepoints. Baseline is shown in blue, midpoint in orange, and endpoint in grey.
C.
D.
Sequences assigned to species
Sequences assigned to level 4 KEGG pathways
p = 0.6304
p = 0.0039*
p = 1.1 e -06*
p = 0.5923
12 months
4 months
9 months
4 months
9 months
12 months
Timepoint
4 months
9 months
12 months
Timepoint

## Slide 5
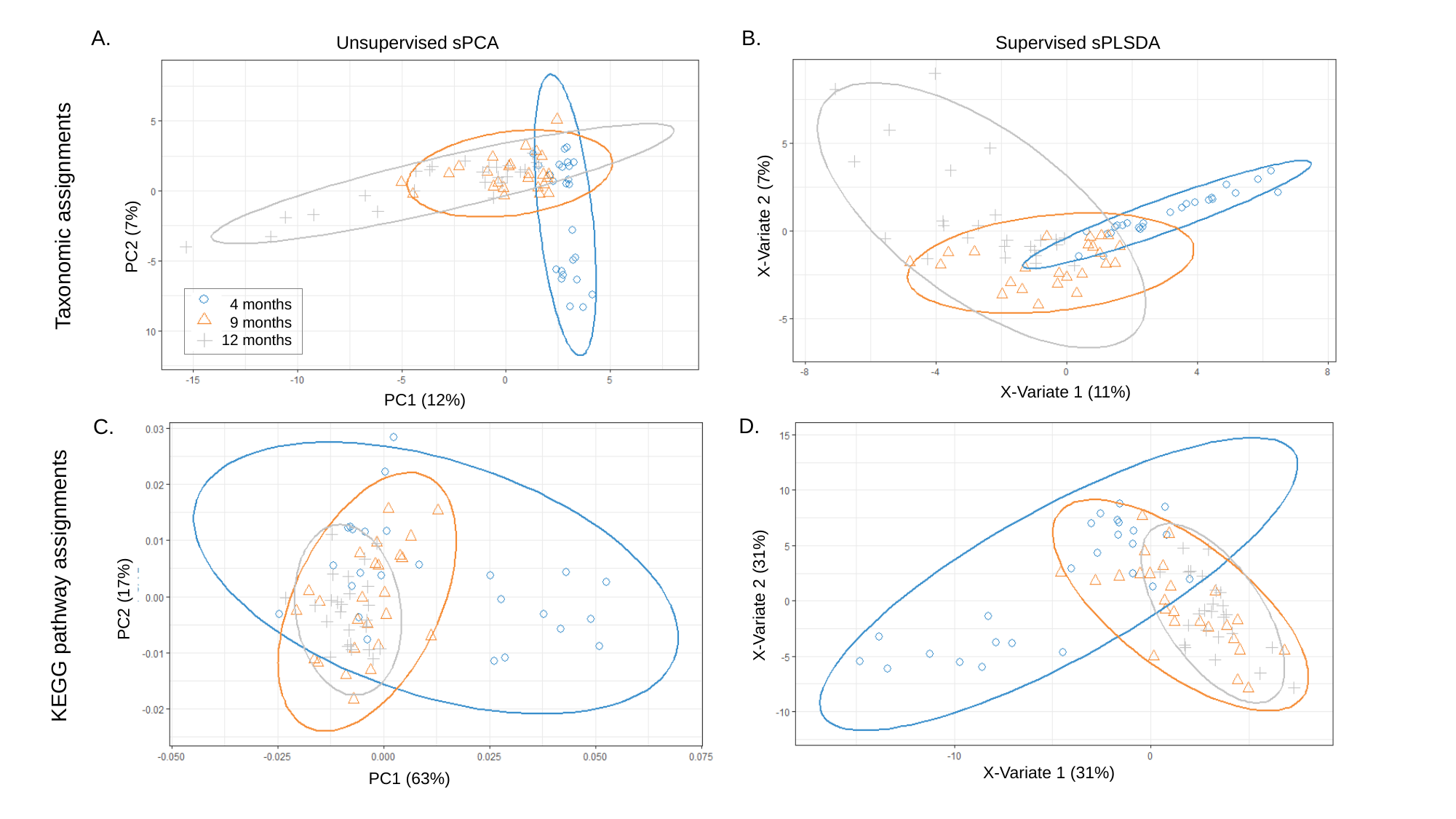

A.
B.
Supervised sPLSDA
Unsupervised sPCA
Taxonomic assignments
X-Variate 2 (7%)
PC2 (7%)
4 months
9 months
12 months
X-Variate 1 (11%)
PC1 (12%)
D.
C.
KEGG pathway assignments
X-Variate 2 (31%)
PC2 (17%)
X-Variate 1 (31%)
PC1 (63%)

## Slide 6
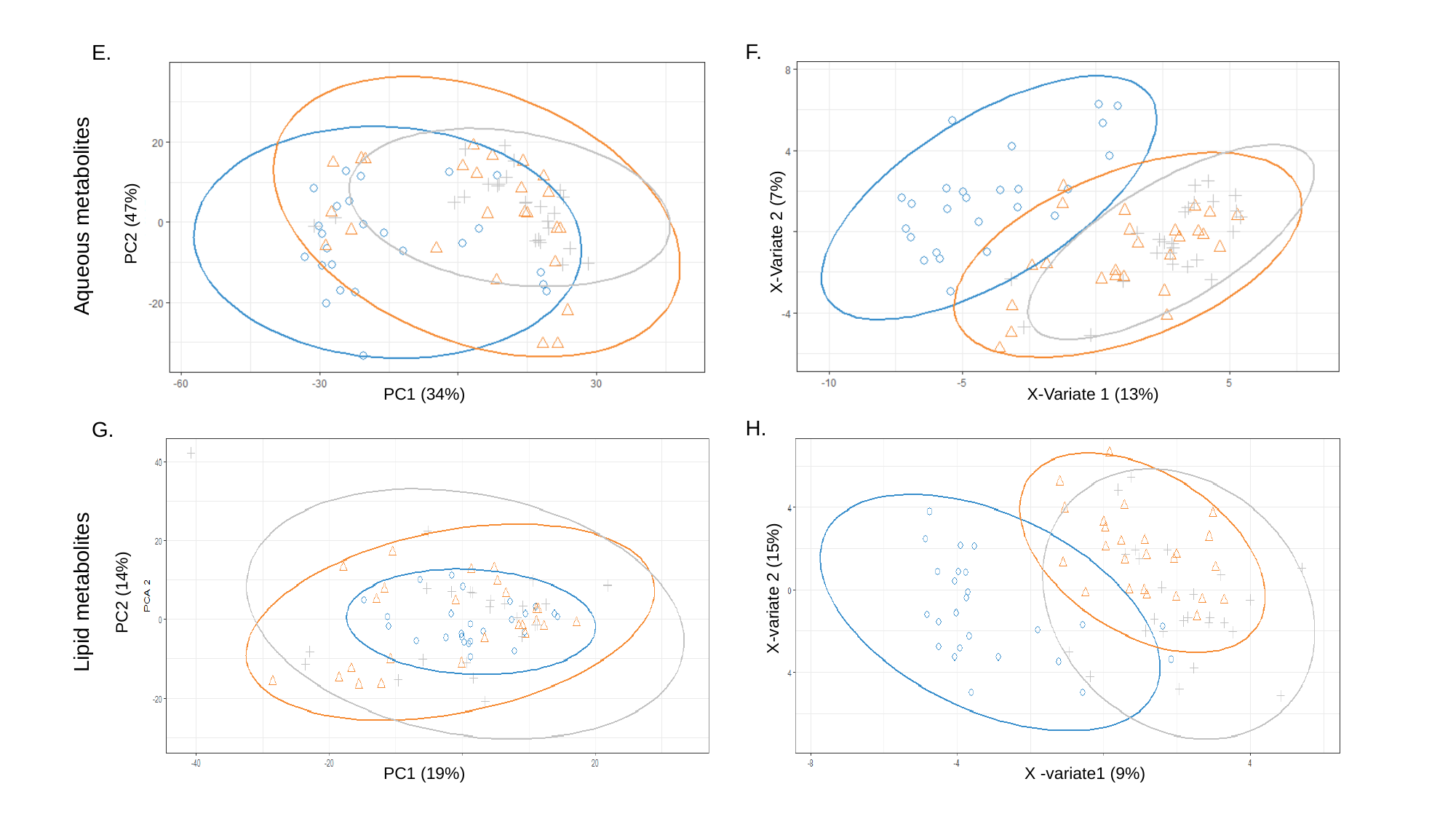

F.
E.
Aqueous metabolites
PC2 (47%)
X-Variate 2 (7%)
PC1 (34%)
X-Variate 1 (13%)
H.
G.
X-variate 2 (15%)
Lipid metabolites
PC2 (14%)
PC1 (19%)
X -variate1 (9%)

## Slide 7
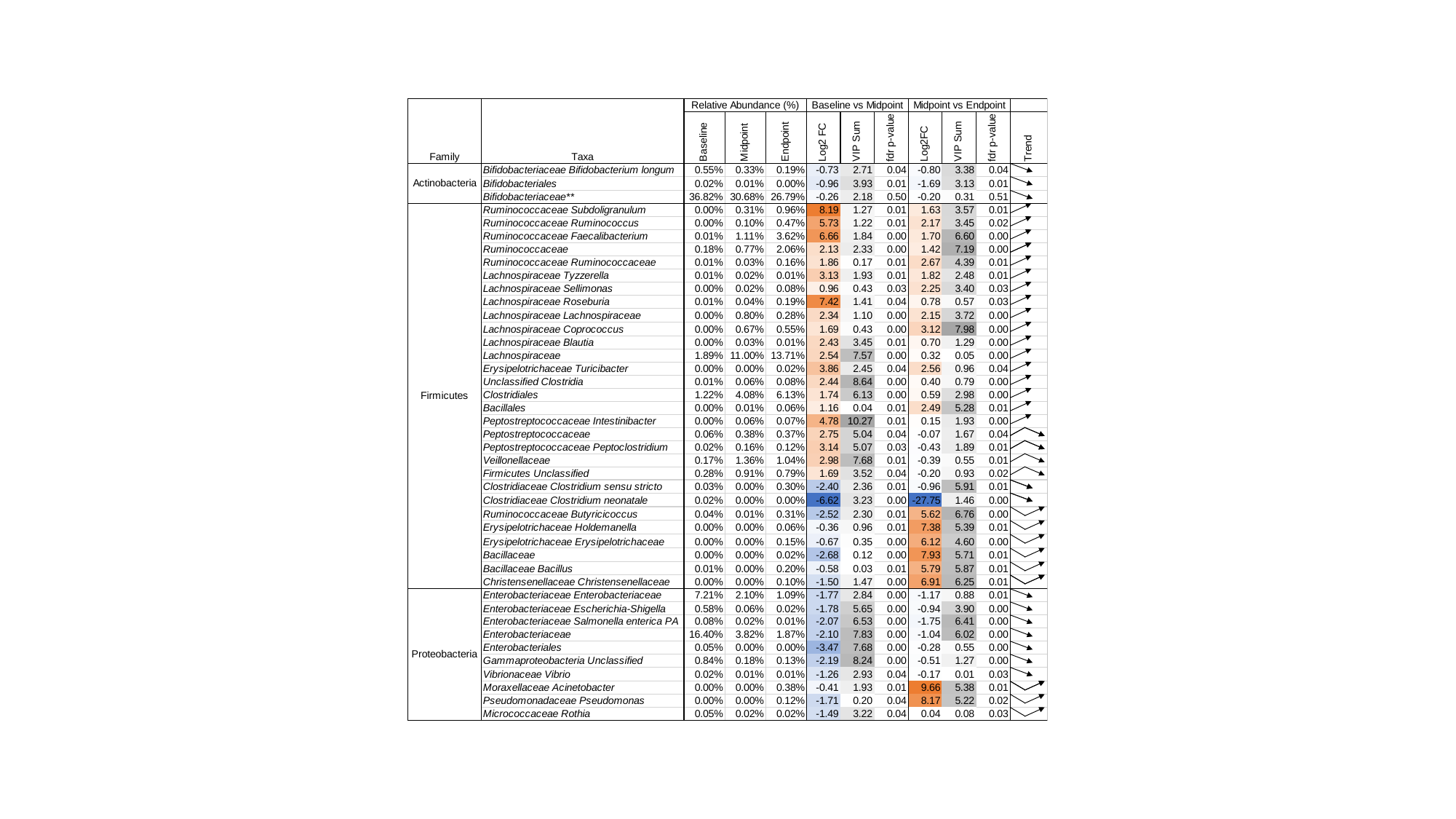

## Slide 8
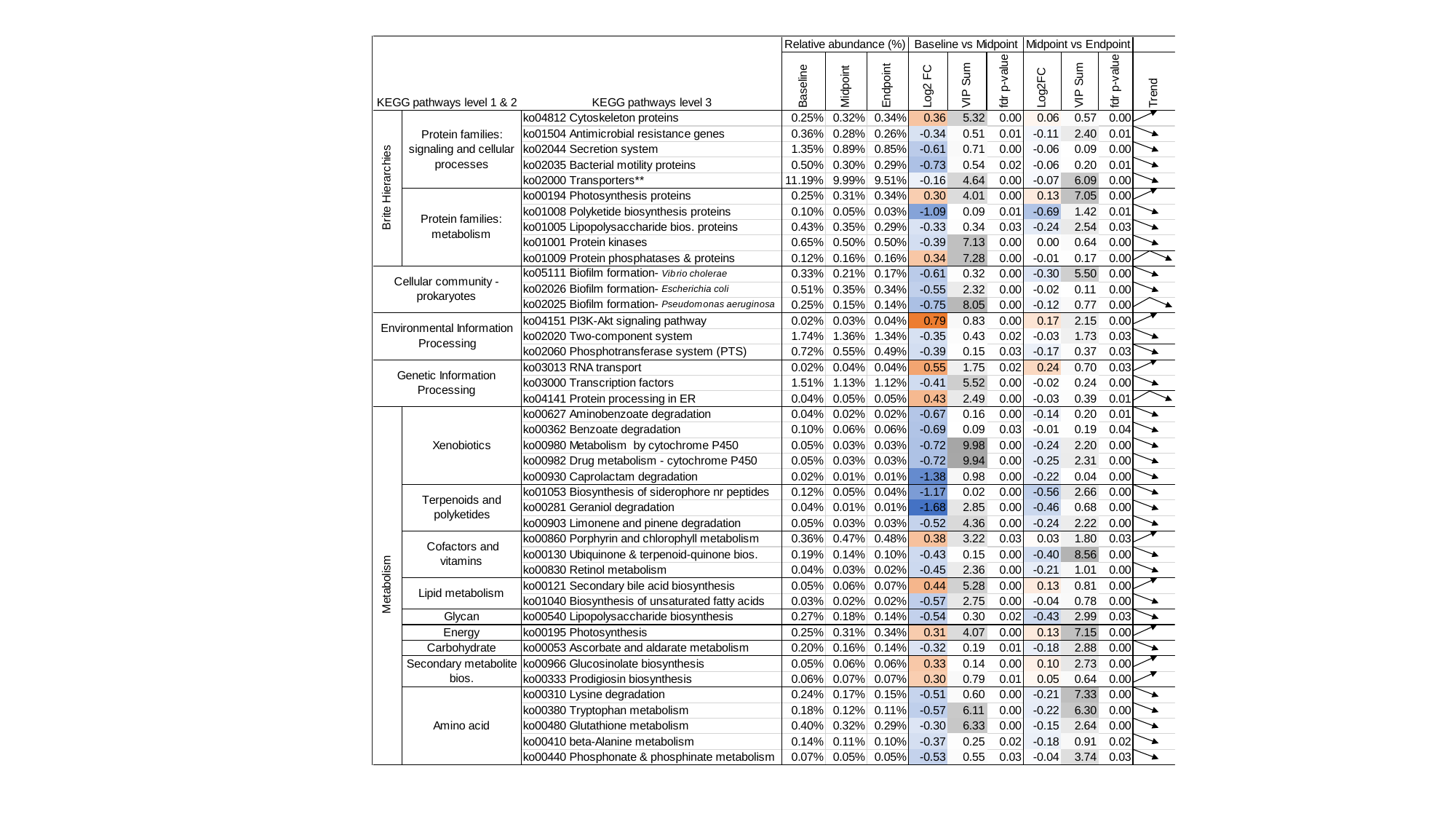

## Slide 9
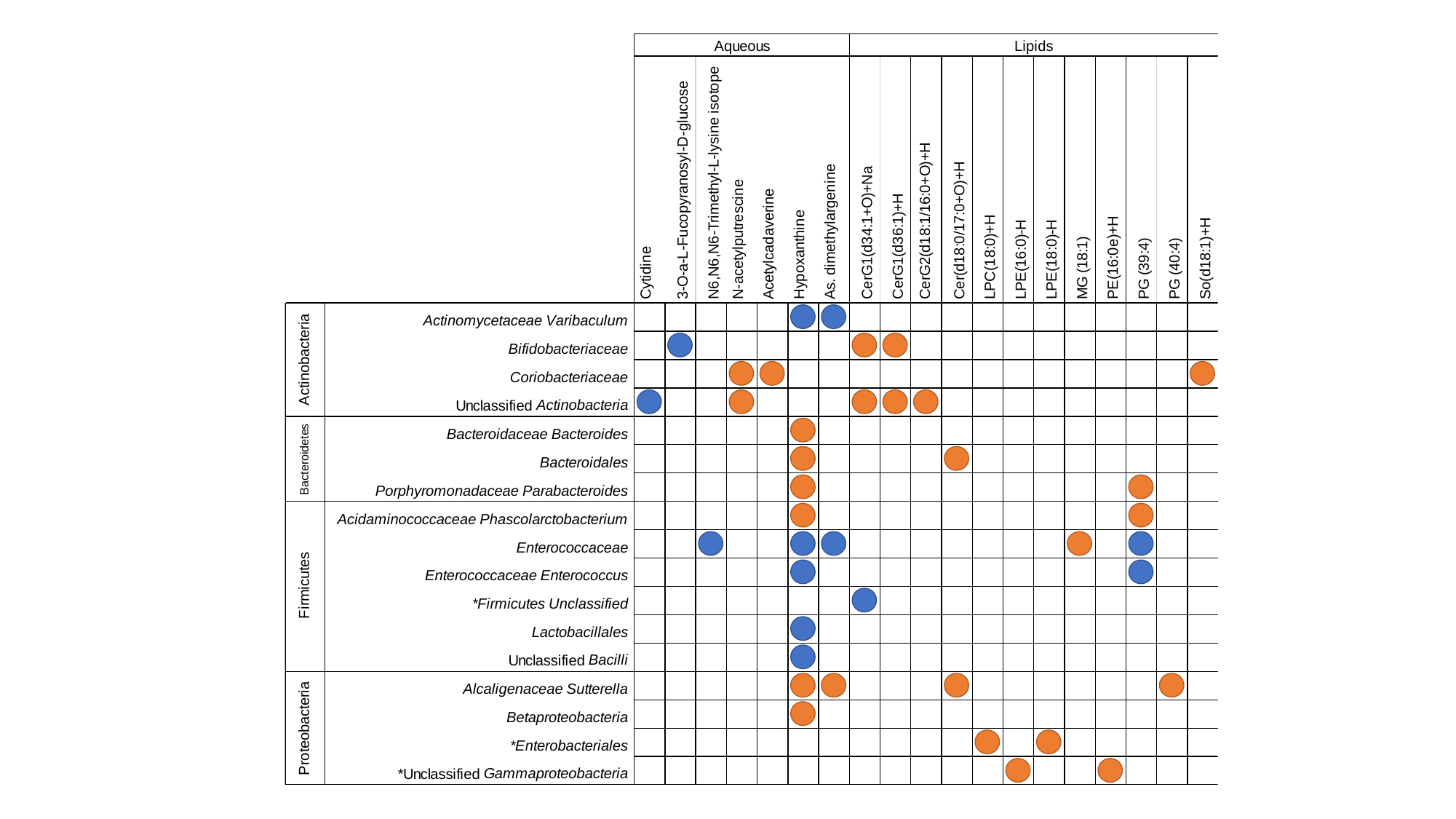

## Slide 10
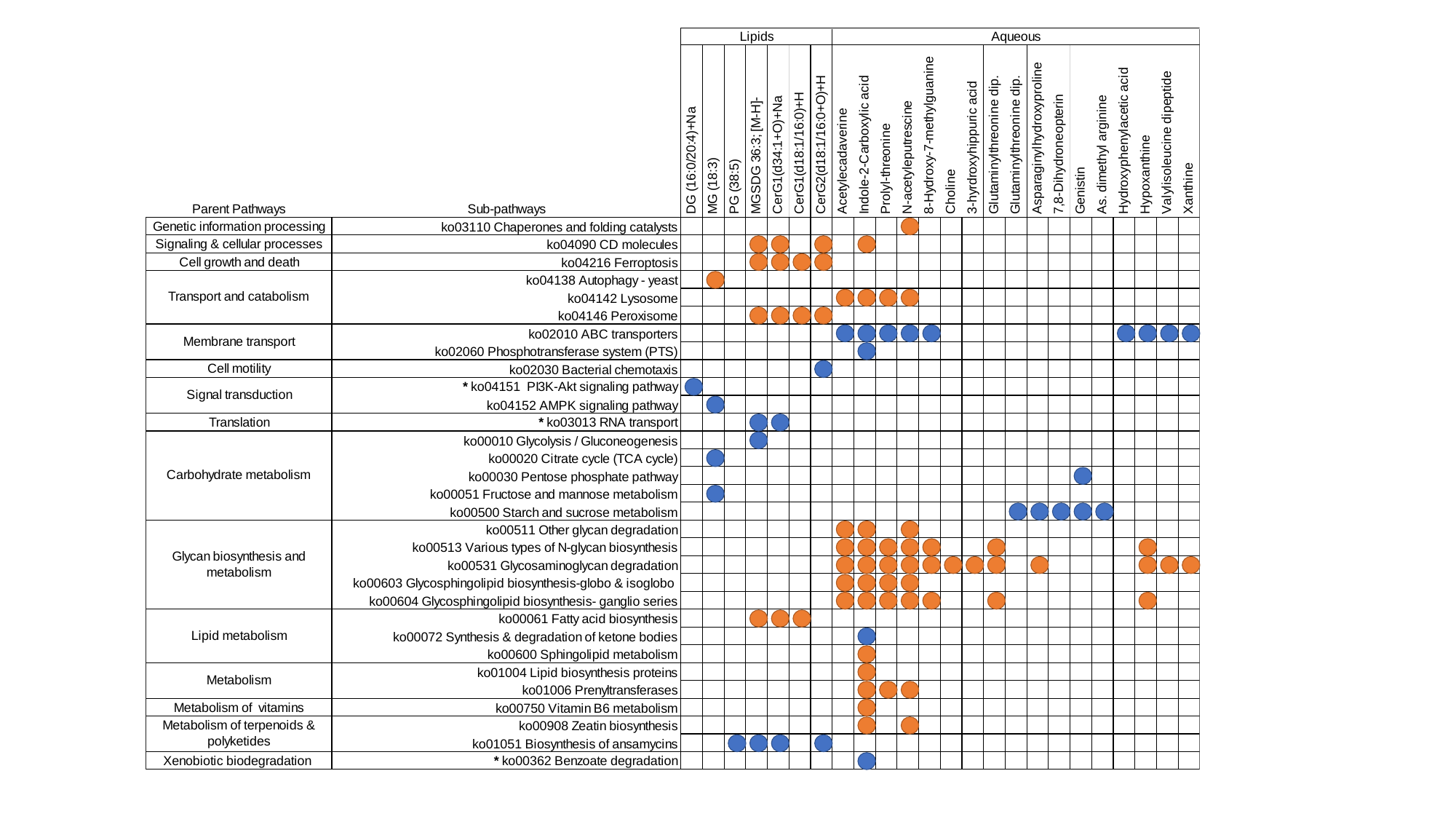

## Slide 11
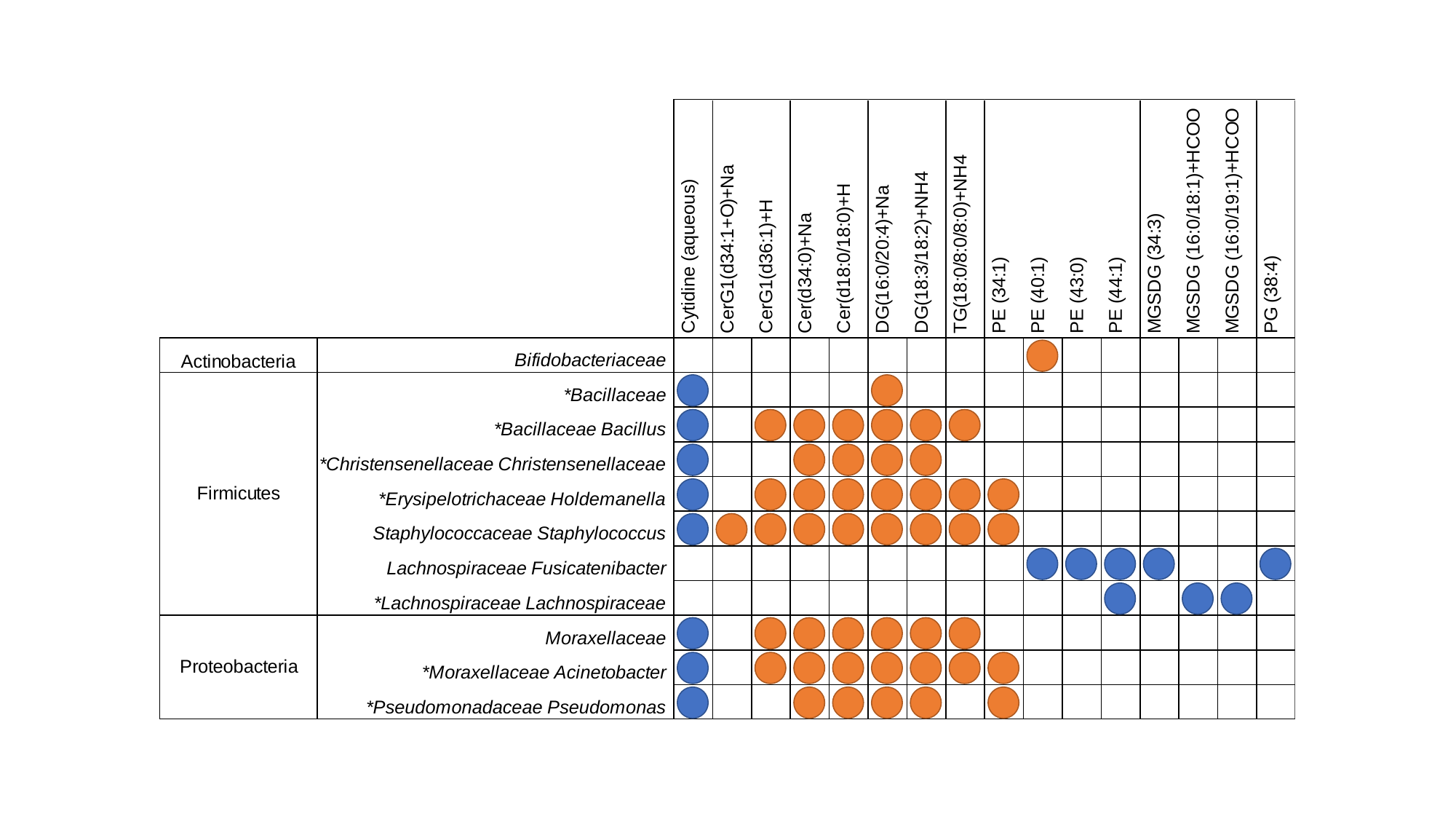

## Slide 12
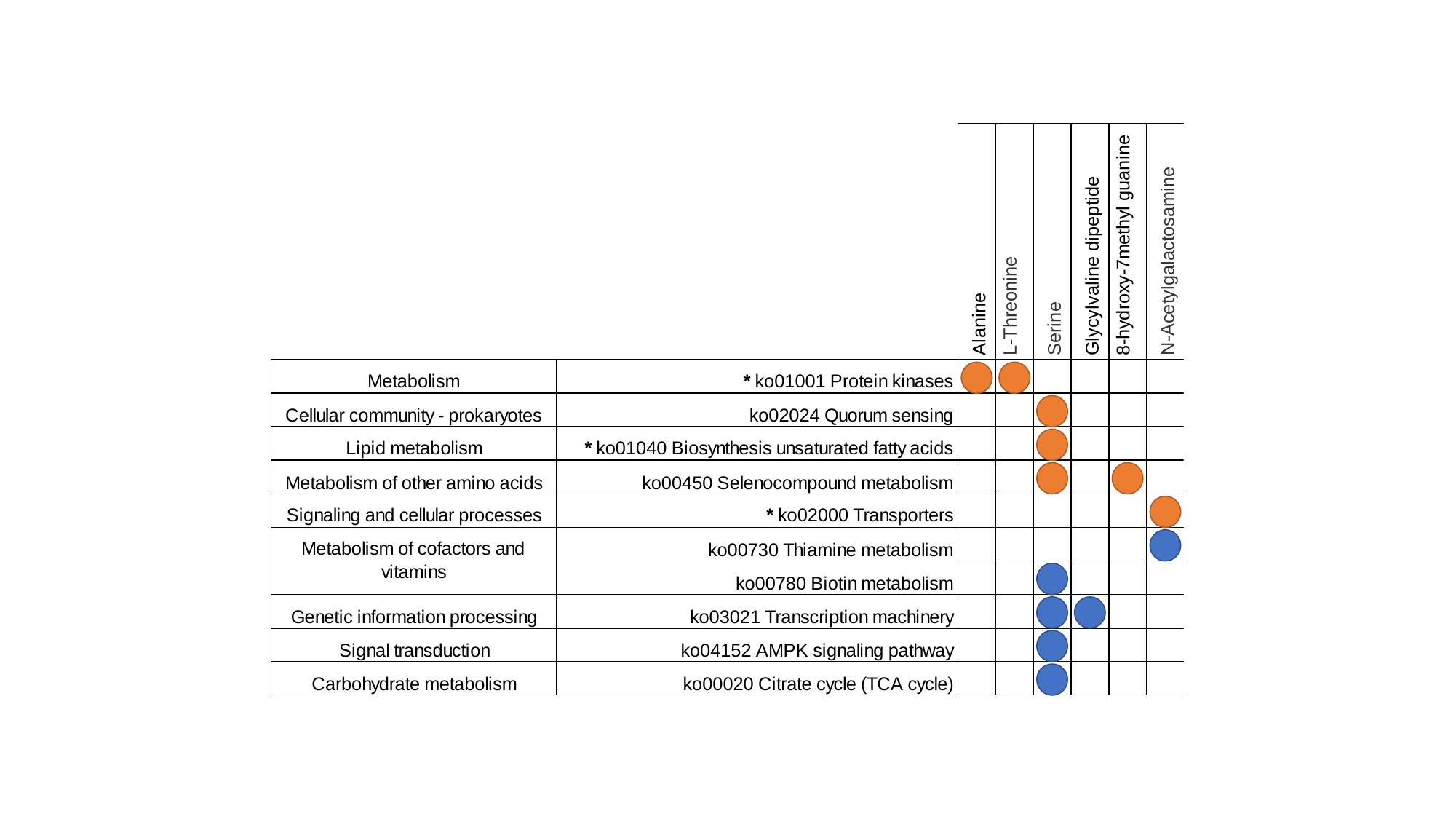

Supplement: S2 File — (ZIP) [file pone.0270213.s002.zip › Final docs/Final formats/TimeseriesFigures.pptx]
