# Supplementary figures and images for: Adaptation of the infant gut microbiome during the complementary feeding transition
Source: PLoS One. 2022 Jul 14;17(7):e0270213. doi: 10.1371/journal.pone.0270213 (PMC9282554; doi:10.1371/journal.pone.0270213)

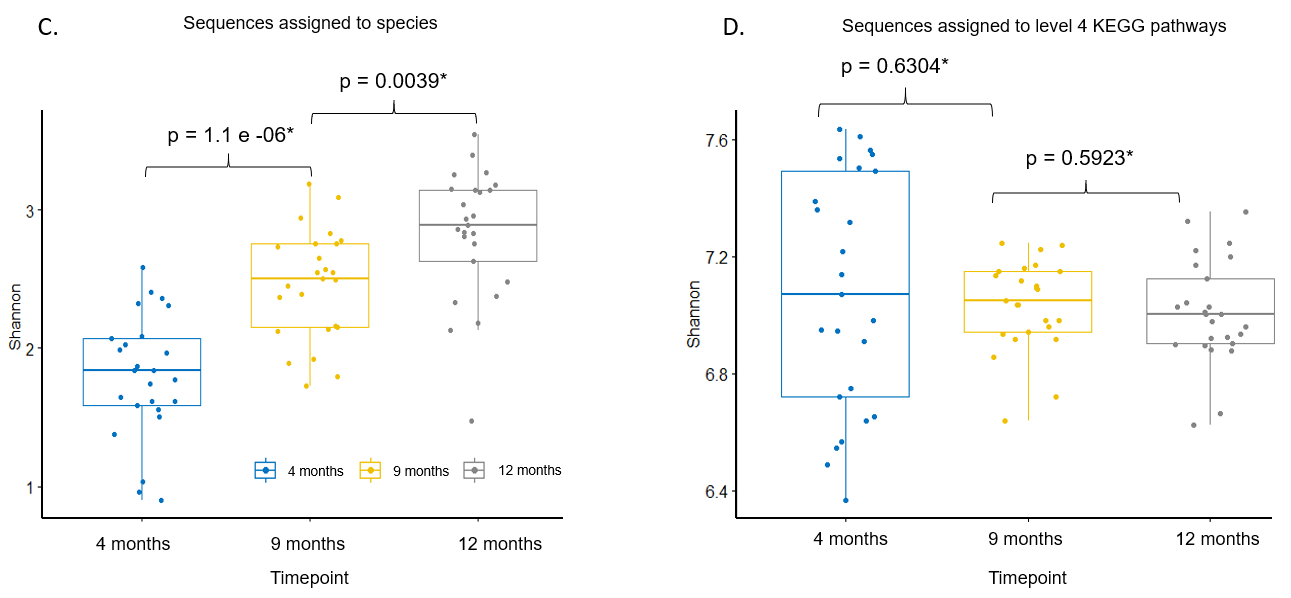

Supplement: S2 File — (ZIP) [file pone.0270213.s002.zip › Final docs/Final formats/AlphaDivTidy.PNG]

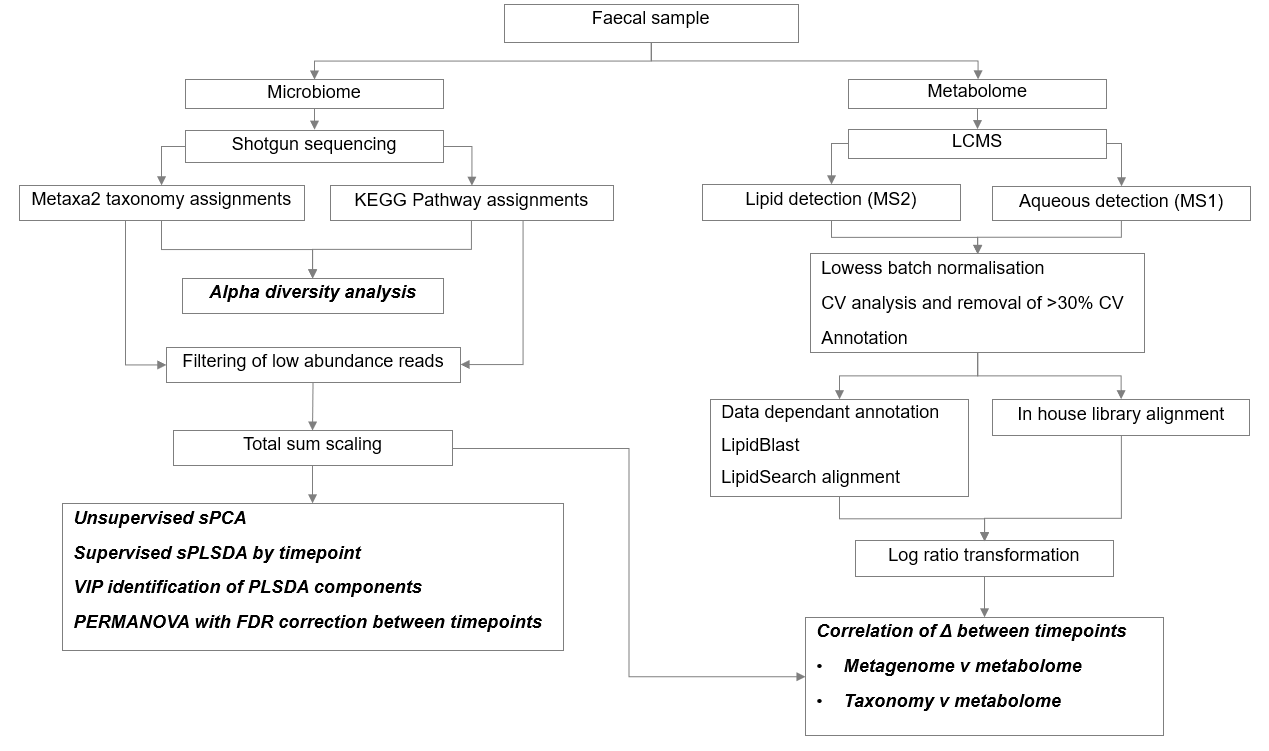

Supplement: S2 File — (ZIP) [file pone.0270213.s002.zip › Final docs/Final formats/AnalyticalApproachDiagram.PNG]

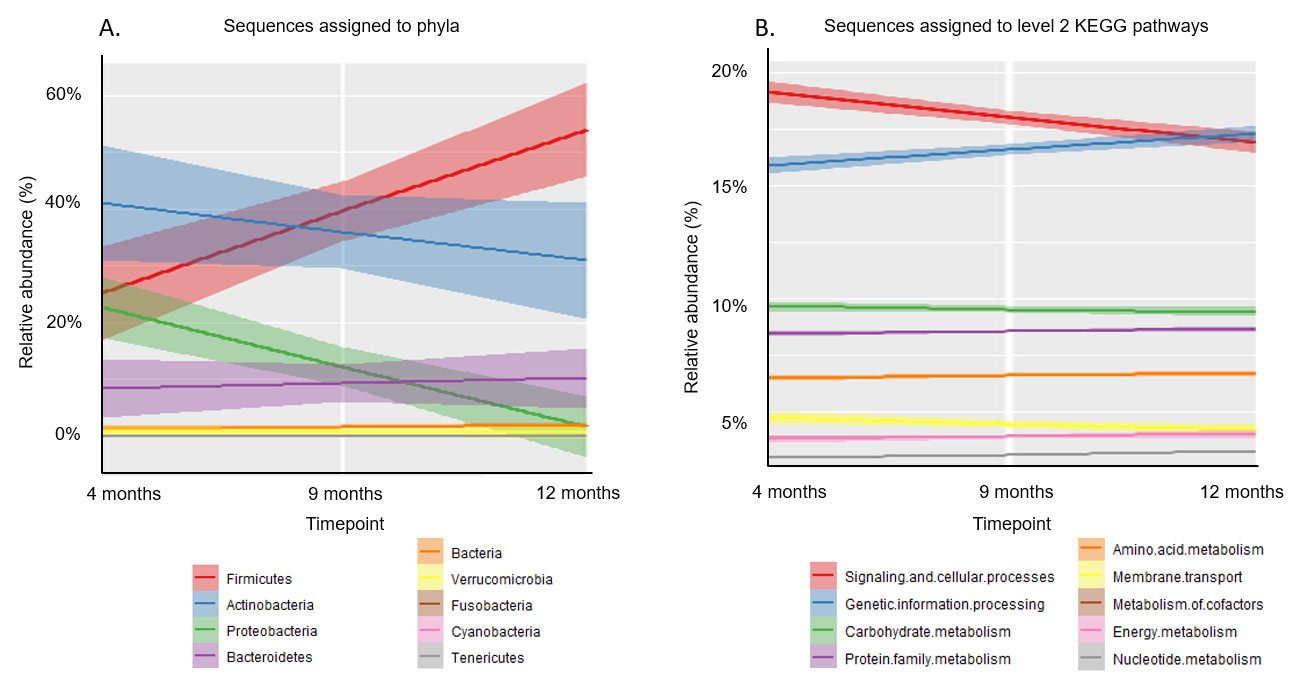

Supplement: S2 File — (ZIP) [file pone.0270213.s002.zip › Final docs/Final formats/BroadTrendsTidy.PNG]

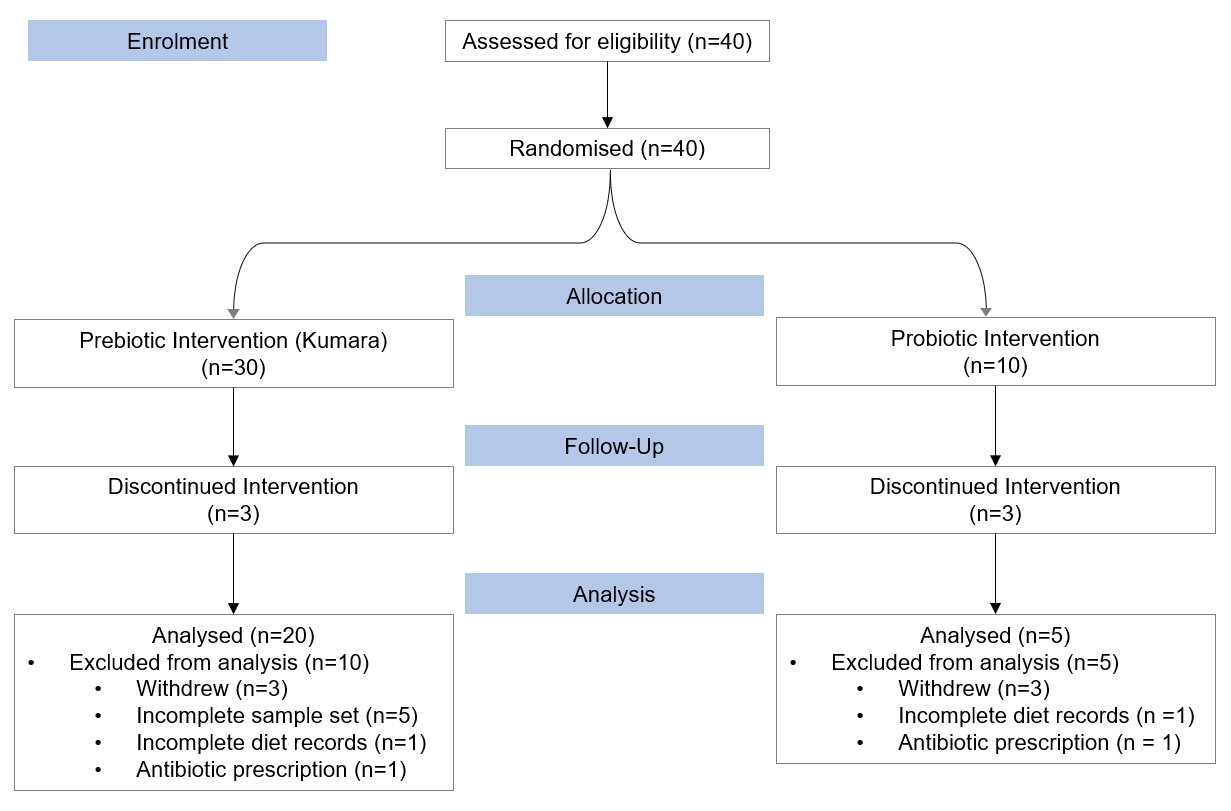

Supplement: S2 File — (ZIP) [file pone.0270213.s002.zip › Final docs/Final formats/CONSORT.PNG]

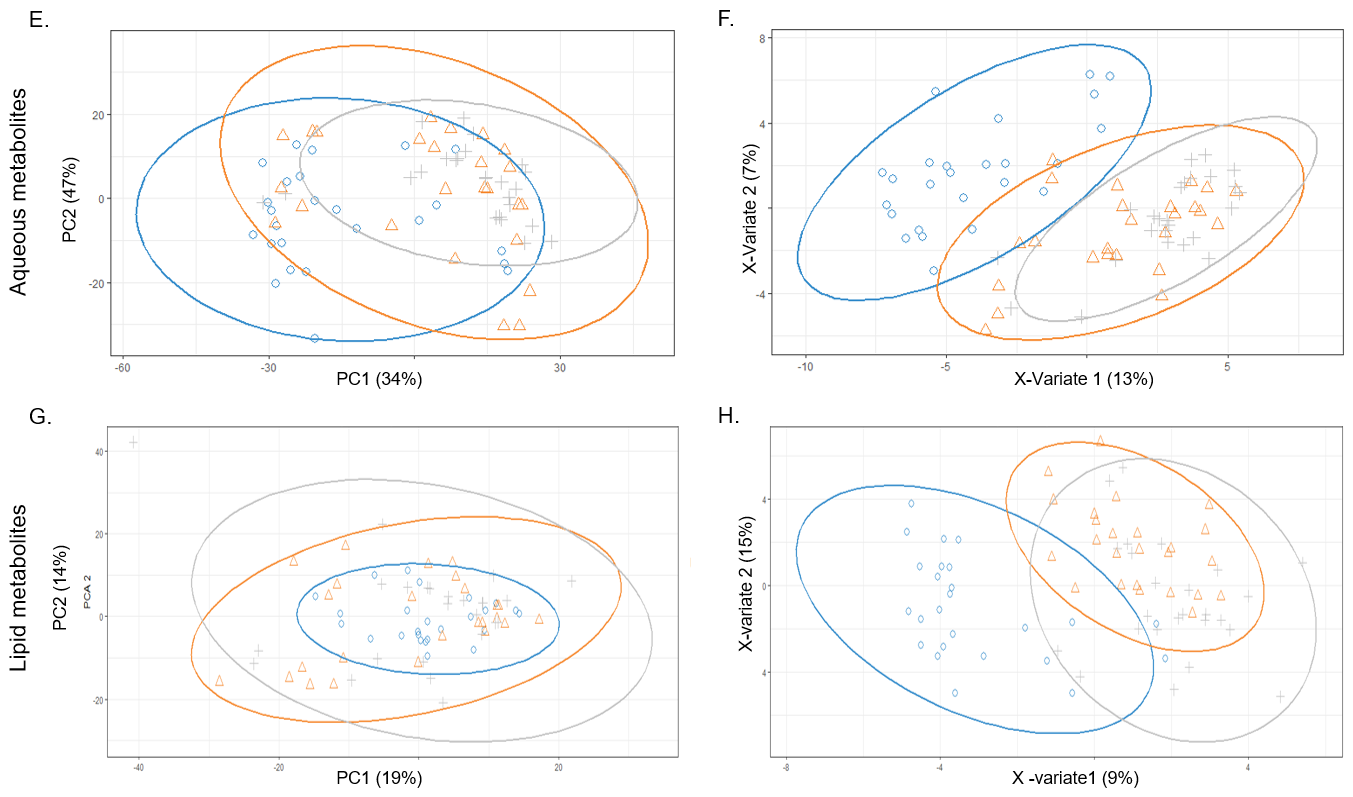

Supplement: S2 File — (ZIP) [file pone.0270213.s002.zip › Final docs/Final formats/PCA_PLSDA_Metabos_Tidy.PNG]

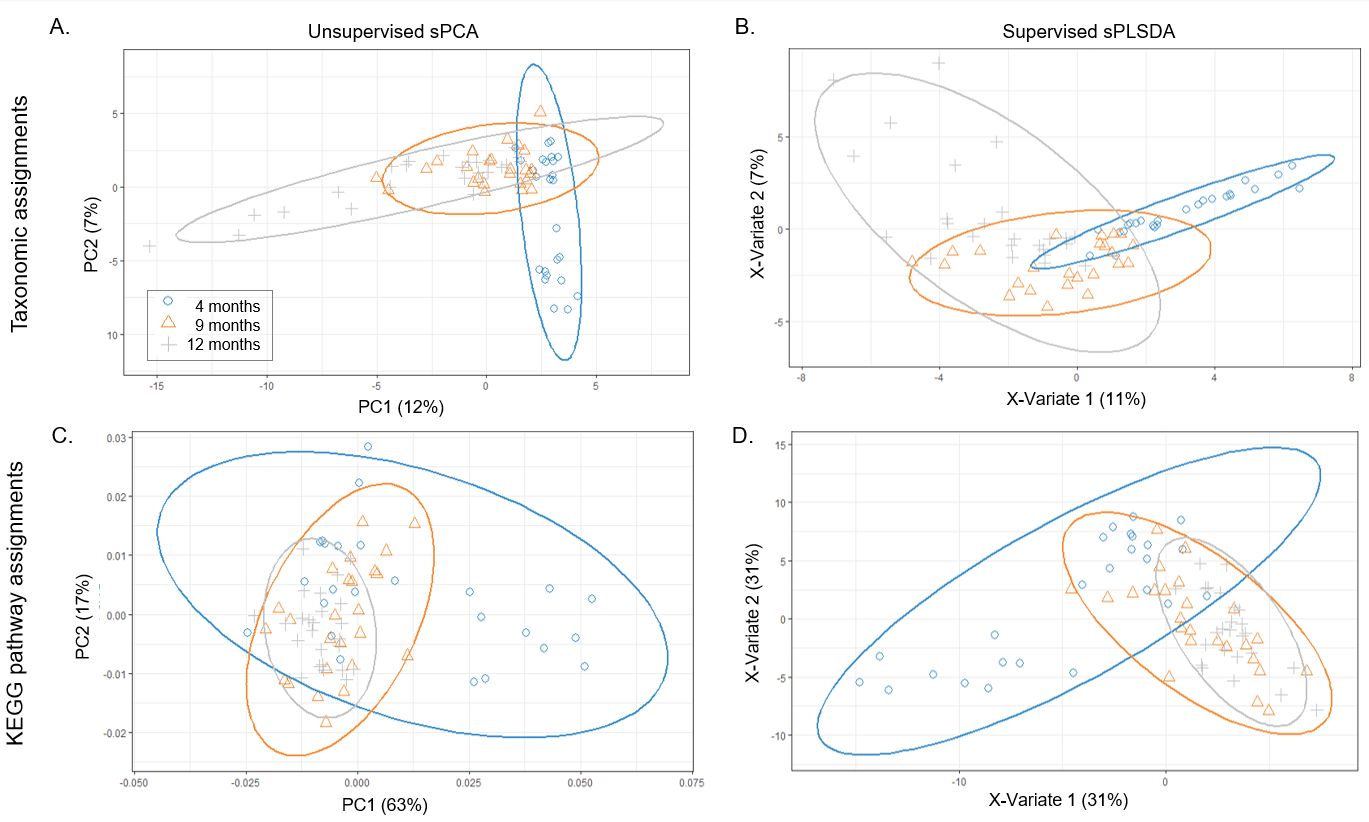

Supplement: S2 File — (ZIP) [file pone.0270213.s002.zip › Final docs/Final formats/PCA_PLSDA_Tidy.PNG]
