# Supplementary material for: Adaptation of the infant gut microbiome during the complementary feeding transition
Source: PLoS One. 2022 Jul 14;17(7):e0270213. doi: 10.1371/journal.pone.0270213 (PMC9282554; doi:10.1371/journal.pone.0270213)
Supplement: S3 File — (DOCX) [file pone.0270213.s003.docx]

**Supplementary Information**

Table S1*. Taxa included in analyses with mean relative abundance at 4, 9 and 12 months of age, as well as Log2 fold change (Log2FC) between timpoints, permanova p values with fdr correction and associated significance (fdr p <0.05) denoted with *.*

| *Taxa* | 4 month mean % | 9 month mean % | 12 month mean % | 4 to 9 month Log2FC | 4 to 9 month Permanova fdr p value | Sig | 9 to 12 month Log2FC | 4 to 9 month permanova fdr p value | Sig |
| --- | --- | --- | --- | --- | --- | --- | --- | --- | --- |
| *Acidaminococcaceae* | 0.0038% | 0.0105% | 0.0083% | 1.4615 | 0.3471 |  | -0.3349 | 0.3288 |  |
| *Acidaminococcaceae Phascolarctobacterium* | 0.0321% | 0.0659% | 0.0471% | 1.0389 | 0.8020 |  | -0.4845 | 0.7944 |  |
| *Actinomycetaceae Actinomyces* | 0.0672% | 0.0763% | 0.0266% | 0.1841 | 0.4141 |  | -1.5208 | 0.3974 |  |
| *Actinomycetaceae Varibaculum* | 0.0215% | 0.0100% | 0.0035% | -1.1001 | 0.3538 |  | -1.5019 | 0.3573 |  |
| *Alcaligenaceae* | 0.0010% | 0.0071% | 0.0151% | 2.7708 | 0.1063 |  | 1.0919 | 0.1278 |  |
| *Alcaligenaceae Parasutterella* | 0.0229% | 0.0520% | 0.3051% | 1.1819 | 0.0499 | * | 2.5521 | 0.0580 |  |
| *Alcaligenaceae Sutterella* | 0.0005% | 0.1260% | 0.0509% | 8.0999 | 0.2356 |  | -1.3088 | 0.2512 |  |
| *Archaea* | 0.0056% | 0.0023% | 0.0114% | -1.2917 | 0.5192 |  | 2.3185 | 0.5236 |  |
| *Bacillaceae* | 0.0005% | 0.0001% | 0.0189% | -2.6849 | 0.0034 | * | 7.9290 | 0.0053 | * |
| *Bacillaceae Bacillus* | 0.0055% | 0.0036% | 0.2023% | -0.5849 | 0.0076 | * | 5.7946 | 0.0053 | * |
| *Bacillales* | 0.0047% | 0.0105% | 0.0588% | 1.1585 | 0.0076 | * | 2.4865 | 0.0053 | * |
| *Bacteroidaceae* | 0.0313% | 0.0237% | 0.0119% | -0.4019 | 0.4804 |  | -0.9987 | 0.4638 |  |
| *Bacteroidaceae Bacteroides* | 5.4570% | 8.7384% | 6.1525% | 0.6793 | 0.6405 |  | -0.5062 | 0.6336 |  |
| *Bacteroidales* | 1.1279% | 2.3327% | 1.6021% | 1.0483 | 0.6087 |  | -0.5420 | 0.6057 |  |
| *Betaproteobacteria* | 0.0035% | 0.0080% | 0.0115% | 1.2051 | 0.1207 |  | 0.5312 | 0.1075 |  |
| *Bifidobacteriaceae* | 36.8184% | 30.6825% | 26.7852% | -0.2630 | 0.5026 |  | -0.1960 | 0.5114 |  |
| *Bifidobacteriaceae Bifidobacterium* | 1.5812% | 1.0421% | 0.9381% | -0.6016 | 0.3140 |  | -0.1516 | 0.3237 |  |
| *Bifidobacteriaceae Bifidobacterium breve* | 0.1246% | 0.0409% | 0.0254% | -1.6078 | 0.1796 |  | -0.6880 | 0.1671 |  |
| *Bifidobacteriaceae Bifidobacterium longum* | 0.5491% | 0.3305% | 0.1896% | -0.7323 | 0.0424 | * | -0.8017 | 0.0362 | * |
| *Bifidobacteriales* | 0.0171% | 0.0088% | 0.0027% | -0.9550 | 0.0120 | * | -1.6850 | 0.0070 | * |
| *Burkholderiales* | 0.0044% | 0.0066% | 0.0054% | 0.5977 | 0.7263 |  | -0.3122 | 0.7210 |  |
| *Christensenellaceae Christensenellaceae* | 0.0022% | 0.0008% | 0.0953% | -1.4994 | 0.0034 | * | 6.9138 | 0.0053 | * |
| *Clostridiaceae Clostridium sensu stricto* | 0.0267% | 0.0004% | 0.3038% | -2.3990 | 0.0093 | * | -0.9623 | 0.0112 | * |
| *Clostridiaceae Sarcina* | 8.2987% | 1.5734% | 0.8076% | -6.2229 | 0.5063 |  | 9.7336 | 0.5236 |  |
| *Clostridiaceae Clostridium neonatale* | 0.0222% | 0.0002% | 0.0000% | -6.6231 | 0.0034 | * | -27.7470 | 0.0034 | * |
| *Clostridiaceae Unclassified* | 0.6958% | 0.0871% | 0.4025% | -2.9978 | 0.3602 |  | 2.2082 | 0.3573 |  |
| *Clostridiales* | 1.2236% | 4.0759% | 6.1331% | 1.7360 | 0.0034 | * | 0.5895 | 0.0034 | * |
| *Clostridiales Family XI Anaerococcus* | 0.0320% | 0.0017% | 0.0020% | -4.2405 | 0.5840 |  | 0.2157 | 0.5631 |  |
| *Coriobacteriaceae* | 0.1520% | 0.2887% | 0.3235% | 0.9260 | 0.6312 |  | 0.1639 | 0.6336 |  |
| *Coriobacteriaceae Atopobium* | 0.0176% | 0.0010% | 0.0008% | -4.1246 | 0.2230 |  | -0.2524 | 0.2159 |  |
| *Coriobacteriaceae Collinsella* | 1.2882% | 0.9326% | 2.4260% | -0.4660 | 0.4804 |  | 1.3793 | 0.4924 |  |
| *Coriobacteriaceae Eggerthella* | 0.0331% | 0.1599% | 0.1201% | 2.2735 | 0.1796 |  | -0.4133 | 0.1671 |  |
| *Desulfovibrionaceae Bilophila* | 0.0032% | 0.0162% | 0.0103% | 2.3525 | 0.5442 |  | -0.6454 | 0.5367 |  |
| *Enterobacteriaceae* | 16.4042% | 3.8237% | 1.8650% | -2.1010 | 0.0034 | * | -1.0357 | 0.0034 | * |
| *Enterobacteriaceae Citrobacter* | 0.0217% | 0.0139% | 0.0064% | -0.6422 | 0.1950 |  | -1.1092 | 0.1900 |  |
| *Enterobacteriaceae Enterobacter* | 0.0155% | 0.0034% | 0.0146% | -2.2022 | 0.6405 |  | 2.1176 | 0.6337 |  |
| *Enterobacteriaceae Enterobacteriaceae bacterium* | 7.2147% | 2.1006% | 1.0923% | -1.7728 | 0.0034 | * | -1.1715 | 0.0083 | * |
| *Enterobacteriaceae Escherichia-Shigella* | 0.5785% | 0.0587% | 0.0221% | -1.7801 | 0.0034 | * | -0.9435 | 0.0034 | * |
| *Enterobacteriaceae Klebsiella* | 0.0129% | 0.0038% | 0.0017% | -3.2998 | 0.2271 |  | -1.4113 | 0.2375 |  |
| *Enterobacteriaceae Salmonella enterica Paratyphi A* | 0.0850% | 0.0202% | 0.0060% | -2.0745 | 0.0034 | * | -1.7520 | 0.0034 | * |
| *Enterobacteriales* | 0.0461% | 0.0041% | 0.0034% | -3.4750 | 0.0034 | * | -0.2763 | 0.0034 | * |
| *Enterococcaceae* | 0.2747% | 0.2852% | 0.1806% | 0.0541 | 0.9860 |  | -0.6589 | 0.9799 |  |
| *Enterococcaceae Enterococcus* | 0.4118% | 0.3675% | 0.2397% | -0.1642 | 0.9017 |  | -0.6167 | 0.9065 |  |
| *Erysipelotrichaceae* | 0.4773% | 0.5348% | 0.5040% | 0.1639 | 1.0000 |  | -0.0856 | 1.0000 |  |
| *Erysipelotrichaceae Coprobacillus* | 0.0000% | 0.0109% | 0.0149% | 33.3396 | 0.2697 |  | 0.4517 | 0.2630 |  |
| *Erysipelotrichaceae Erysipelatoclostridium* | 2.6876% | 0.4544% | 0.4396% | -2.5643 | 0.3818 |  | -0.0477 | 0.3846 |  |
| *Erysipelotrichaceae Erysipelotrichaceae* | 0.0035% | 0.0022% | 0.1512% | -0.6710 | 0.0034 | * | 6.1158 | 0.0034 | * |
| *Erysipelotrichaceae Faecalitalea* | 0.0224% | 0.0034% | 0.0102% | -2.7086 | 1.0000 |  | 1.5679 | 1.0000 |  |
| *Erysipelotrichaceae Holdemanella* | 0.0005% | 0.0004% | 0.0626% | -0.3585 | 0.0107 | * | 7.3847 | 0.0053 | * |
| *Erysipelotrichaceae Turicibacter* | 0.0002% | 0.0026% | 0.0156% | 3.8577 | 0.0370 | * | 2.5619 | 0.0438 | * |
| *Firmicutes Unclassified* | 0.2814% | 0.9073% | 0.7906% | 1.6892 | 0.0397 | * | -0.1987 | 0.0211 | * |
| *Fusobacteriaceae Fusobacterium* | 0.0130% | 0.0445% | 0.0714% | 1.7787 | 0.5063 |  | 0.6830 | 0.5114 |  |
| *Gammaproteobacteria Unclassified* | 0.8406% | 0.1836% | 0.1291% | -2.1944 | 0.0034 | * | -0.5084 | 0.0034 | * |
| *Lachnospiraceae* | 1.8861% | 11.0013% | 13.7104% | 2.5442 | 0.0034 | * | 0.3176 | 0.0034 | * |
| *Lachnospiraceae Anaerostipes* | 0.0039% | 0.0204% | 0.0164% | 7.3603 | 0.0580 |  | -0.3030 | 0.0632 |  |
| *Lachnospiraceae Blautia* | 0.0000% | 0.0256% | 0.0110% | 2.4293 | 0.0093 | * | 0.7027 | 0.0034 | * |
| *Lachnospiraceae Coprococcus* | 0.0041% | 0.6748% | 0.5470% | 1.6946 | 0.0034 | * | 3.1244 | 0.0034 | * |
| *Lachnospiraceae Dorea* | 0.5486% | 2.9549% | 4.8092% | 7.9527 | 0.3918 |  | -0.5741 | 0.4079 |  |
| *Lachnospiraceae Eubacterium rectale* | 0.0022% | 0.0072% | 0.0627% | 2.4002 | 0.2272 |  | -0.3121 | 0.2222 |  |
| *Lachnospiraceae Fusicatenibacter* | 0.0008% | 0.2035% | 0.1367% | 4.8055 | 0.2126 |  | 0.6406 | 0.2211 |  |
| *Lachnospiraceae Hungatella* | 0.0034% | 0.0952% | 0.1484% | 1.8439 | 0.1947 |  | -0.9112 | 0.1828 |  |
| *Lachnospiraceae Lachnoclostridium* | 0.0213% | 0.0763% | 0.0406% | 0.4701 | 0.6120 |  | 0.4872 | 0.6057 |  |
| *Lachnospiraceae Lachnospira* | 0.2689% | 0.3725% | 0.5221% | 8.2041 | 0.8020 |  | -1.4999 | 0.8116 |  |
| *Lachnospiraceae Lachnospiraceae* | 0.0027% | 0.7969% | 0.2818% | 2.3385 | 0.0034 | * | 2.1493 | 0.0034 | * |
| *Lachnospiraceae Roseburia* | 0.0084% | 0.0426% | 0.1891% | 7.4194 | 0.0424 | * | 0.7754 | 0.0329 | * |
| *Lachnospiraceae Ruminococcus gauvreauii* | 0.0023% | 0.3945% | 0.6753% | 34.5779 | 0.6259 |  | -1.2172 | 0.6057 |  |
| *Lachnospiraceae Ruminococcus gnavus* | 0.0015% | 0.0029% | 0.0139% | 0.3362 | 0.9809 |  | -0.6246 | 0.9799 |  |
| *Lachnospiraceae Sellimonas* | 0.0026% | 0.0227% | 0.0800% | 0.9639 | 0.0334 | * | 2.2453 | 0.0265 | * |
| *Lachnospiraceae Tyzzerella* | 0.0144% | 0.0182% | 0.0118% | 3.1253 | 0.0135 | * | 1.8172 | 0.0083 | * |
| *Lactobacillaceae* | 0.0016% | 0.0319% | 0.0059% | 4.3464 | 0.2697 |  | -2.4252 | 0.2630 |  |
| *Lactobacillaceae Lactobacillus* | 0.3914% | 1.9382% | 1.0520% | 2.3078 | 0.3538 |  | -0.8815 | 0.3448 |  |
| *Lactobacillaceae Lactobacillus rhamnosus* | 0.0045% | 0.0154% | 0.0013% | 1.7712 | 0.0819 |  | -3.5513 | 0.0819 |  |
| *Lactobacillales* | 0.1751% | 0.2601% | 0.1748% | 0.5708 | 0.6745 |  | -0.5732 | 0.6650 |  |
| *Micrococcaceae Rothia* | 0.0469% | 0.0167% | 0.0171% | -1.4930 | 0.0370 | * | 0.0396 | 0.0336 | * |
| *Moraxellaceae* | 0.0079% | 0.0017% | 0.0189% | -2.1841 | 0.0974 |  | 3.4513 | 0.0974 |  |
| *Moraxellaceae Acinetobacter* | 0.0006% | 0.0005% | 0.3753% | -0.4081 | 0.0076 | * | 9.6612 | 0.0100 | * |
| *Negativicutes* | 0.0025% | 0.0090% | 0.0090% | 1.8366 | 0.1947 |  | 0.0051 | 0.1908 |  |
| *Pasteurellaceae* | 0.0114% | 0.0911% | 0.0490% | 2.9989 | 0.1950 |  | -0.8928 | 0.1957 |  |
| *Pasteurellaceae Haemophilus* | 0.0242% | 0.2433% | 0.1323% | 3.3310 | 0.2199 |  | -0.8784 | 0.1900 |  |
| *Peptostreptococcaceae* | 0.0571% | 0.3842% | 0.3655% | 2.7514 | 0.0416 | * | -0.0718 | 0.0357 | * |
| *Peptostreptococcaceae Intestinibacter* | 0.0023% | 0.0636% | 0.0707% | 4.7760 | 0.0076 | * | 0.1532 | 0.0034 | * |
| *Peptostreptococcaceae Peptoclostridium* | 0.0179% | 0.1579% | 0.1176% | 3.1388 | 0.0254 | * | -0.4256 | 0.0112 | * |
| *Porphyromonadaceae Barnesiella* | 0.0005% | 0.0052% | 0.0152% | 3.4959 | 0.2126 |  | 1.5567 | 0.2159 |  |
| *Porphyromonadaceae Parabacteroides* | 0.2242% | 0.5905% | 0.2460% | 1.3969 | 0.7753 |  | -1.2635 | 0.7944 |  |
| *Prevotellaceae* | 0.0029% | 0.0524% | 0.0318% | 4.1959 | 0.6394 |  | -0.7212 | 0.6530 |  |
| *Prevotellaceae Prevotella* | 0.0130% | 0.5417% | 0.4308% | 5.3793 | 0.6745 |  | -0.3305 | 0.6563 |  |
| *Pseudomonadaceae Pseudomonas* | 0.0014% | 0.0004% | 0.1248% | -1.7064 | 0.0376 | * | 8.1666 | 0.0239 | * |
| *Rikenellaceae Alistipes* | 0.0040% | 0.1023% | 0.0856% | 4.6916 | 0.2733 |  | -0.2570 | 0.2630 |  |
| *Ruminococcaceae* | 0.1751% | 0.7676% | 2.0576% | 2.1323 | 0.0034 | * | 1.4226 | 0.0034 | * |
| *Ruminococcaceae Butyricicoccus* | 0.0355% | 0.0062% | 0.3053% | -2.5212 | 0.0064 | * | 5.6235 | 0.0034 | * |
| *Ruminococcaceae Faecalibacterium* | 0.0110% | 1.1116% | 3.6189% | 6.6572 | 0.0034 | * | 1.7028 | 0.0034 | * |
| *Ruminococcaceae Flavonifractor* | 0.0477% | 0.0710% | 0.1081% | 0.5746 | 0.3454 |  | 0.6057 | 0.3448 |  |
| *Ruminococcaceae Ruminiclostridium* | 0.0102% | 0.0501% | 0.0990% | 2.2917 | 0.2272 |  | 0.9813 | 0.2375 |  |
| *Ruminococcaceae Ruminococcaceae* | 0.0069% | 0.0252% | 0.1599% | 1.8634 | 0.0150 | * | 2.6658 | 0.0070 | * |
| *Ruminococcaceae Ruminococcus* | 0.0019% | 0.1035% | 0.4667% | 5.7312 | 0.0120 | * | 2.1728 | 0.0163 | * |
| *Ruminococcaceae Subdoligranulum* | 0.0011% | 0.3104% | 0.9611% | 8.1873 | 0.0107 | * | 1.6305 | 0.0070 | * |
| *Selenomonadales* | 0.0138% | 0.0642% | 0.0250% | 2.2210 | 0.2667 |  | -1.3602 | 0.2630 |  |
| *Staphylococcaceae Staphylococcus* | 0.0353% | 0.0199% | 0.1333% | -0.8276 | 0.0819 |  | 2.7459 | 0.0760 |  |
| *Streptococcaceae* | 0.0056% | 0.0139% | 0.0150% | 1.3124 | 0.5840 |  | 0.1060 | 0.5876 |  |
| *Streptococcaceae Lactococcus* | 0.0007% | 0.3313% | 0.0399% | 8.8431 | 0.9550 |  | -3.0553 | 0.9601 |  |
| *Streptococcaceae Streptococcus* | 0.7379% | 1.3085% | 1.8564% | 0.8264 | 0.4804 |  | 0.5046 | 0.4235 |  |
| *Streptomycetaceae Streptomyces* | 0.1083% | 0.0975% | 0.0857% | -0.1517 | 0.7260 |  | -0.1851 | 0.7405 |  |
| *Unclassified Actinobacteria* | 0.9834% | 0.7191% | 0.6182% | -0.4517 | 0.2697 |  | -0.2181 | 0.2667 |  |
| *Unclassified Bacilli* | 0.0284% | 0.0479% | 0.0533% | 0.7579 | 0.3602 |  | 0.1514 | 0.3714 |  |
| *Unclassified Bacteria* | 1.4164% | 1.8471% | 2.0130% | 0.3830 | 0.2939 |  | 0.1241 | 0.2866 |  |
| *Unclassified Bacteroidetes* | 0.0180% | 0.0501% | 0.0379% | 1.4753 | 0.5154 |  | -0.4004 | 0.5066 |  |
| *Unclassified Clostridia* | 0.0107% | 0.0582% | 0.0768% | 2.4373 | 0.0034 | * | 0.3999 | 0.0034 | * |
| *Unclassified Proteobacteria* | 0.0418% | 0.0144% | 0.0258% | -1.5324 | 0.0469 | * | 0.8351 | 0.0552 |  |
| *Unknown* | 0.1270% | 0.0676% | 0.1747% | -0.9107 | 0.3650 |  | 1.3698 | 0.3573 |  |
| *Veillonellaceae* | 0.1729% | 1.3633% | 1.0428% | 2.9788 | 0.0076 | * | -0.3867 | 0.0083 | * |
| *Veillonellaceae Dialister* | 0.0019% | 0.1673% | 0.3750% | 6.4474 | 0.2697 |  | 1.1644 | 0.2630 |  |
| *Veillonellaceae Megamonas* | 0.0466% | 3.0303% | 0.4491% | 6.0236 | 0.3575 |  | -2.7542 | 0.3573 |  |
| *Veillonellaceae Megasphaera* | 0.0196% | 0.4455% | 1.0922% | 4.5031 | 0.2491 |  | 1.2937 | 0.2630 |  |
| *Veillonellaceae Veillonella* | 3.5527% | 6.0813% | 3.9140% | 0.7755 | 0.3471 |  | -0.6357 | 0.3448 |  |
| *Verrucomicrobiaceae Akkermansia* | 1.0222% | 0.4354% | 0.9406% | -1.2312 | 0.9550 |  | 1.1111 | 0.9601 |  |
| *Vibrionaceae Vibrio* | 0.0208% | 0.0087% | 0.0077% | -1.2573 | 0.0370 | * | -0.1691 | 0.0322 | * |

Table S2*. KEGG pathways included in analyses with mean relative abundance at 4, 9 and 12 months of age, as well as Log2 fold change (Log2FC) between timpoints, permanova p values with fdr correction and associated significance (fdr p <0.05) denoted with *.*

| KEGG pathway | 4 month mean % | 9 month mean % | 12 month mean % | 4 to 9 month Log2FC | 4 to 9 month Permanova fdr p value | Sig | 9 to 12 month Log2FC | 4 to 9 month permanova fdr p value | Sig |
| --- | --- | --- | --- | --- | --- | --- | --- | --- | --- |
| ko03000.Transcription.factors. | 1.5062% | 1.1316% | 1.1150% | -0.4125 | 0.0017 | * | -0.0213 | 0.0018 | * |
| ko03009.Ribosome.biogenesis. | 1.2119% | 1.2624% | 1.3314% | 0.0589 | 0.0017 | * | 0.0768 | 0.0018 | * |
| ko03011.Ribosome. | 0.8666% | 0.9639% | 0.9816% | 0.1535 | 0.0244 | * | 0.0263 | 0.0330 | * |
| ko03012.Translation.factors. | 0.5309% | 0.6043% | 0.6222% | 0.1866 | 0.0017 | * | 0.0423 | 0.0018 | * |
| ko03016.Transfer.RNA.biogenesis. | 2.3256% | 2.5929% | 2.6611% | 0.1570 | 0.0017 | * | 0.0375 | 0.0018 | * |
| ko03019.Messenger.RNA.biogenesis. | 0.7728% | 0.8344% | 0.8673% | 0.1107 | 0.0017 | * | 0.0558 | 0.0018 | * |
| ko03021.Transcription.machinery. | 0.6830% | 0.7984% | 0.8172% | 0.2252 | 0.0017 | * | 0.0337 | 0.0018 | * |
| ko03029.Mitochondrial.biogenesis. | 1.1648% | 1.3270% | 1.3724% | 0.1881 | 0.0017 | * | 0.0485 | 0.0018 | * |
| ko03032.DNA.replication.proteins. | 1.3743% | 1.5738% | 1.6622% | 0.1955 | 0.0017 | * | 0.0788 | 0.0018 | * |
| ko03036.Chromosome.and.associated.proteins. | 1.2516% | 1.3303% | 1.3509% | 0.0880 | 0.0017 | * | 0.0222 | 0.0018 | * |
| ko03051.Proteasome. | 0.1056% | 0.1108% | 0.1136% | 0.0699 | 0.8874 |  | 0.0360 | 0.8766 |  |
| ko03110.Chaperones.and.folding.catalysts. | 0.9103% | 0.9819% | 0.9986% | 0.1092 | 0.0172 | * | 0.0244 | 0.0113 | * |
| ko03400.DNA.repair.and.recombination.proteins. | 3.5172% | 3.9165% | 4.0279% | 0.1551 | 0.0044 | * | 0.0405 | 0.0031 | * |
| ko04131.Membrane.trafficking. | 0.3421% | 0.3661% | 0.3472% | 0.0977 | 0.7528 |  | -0.0766 | 0.7594 |  |
| ko00194.Photosynthesis.proteins. | 0.2507% | 0.3088% | 0.3377% | 0.3009 | 0.0017 | * | 0.1291 | 0.0018 | * |
| ko01001.Protein.kinases. | 0.6539% | 0.5001% | 0.4998% | -0.3868 | 0.0017 | * | -0.0007 | 0.0018 | * |
| ko01002.Peptidases. | 1.9755% | 2.0491% | 2.0921% | 0.0528 | 0.0105 | * | 0.0300 | 0.0066 | * |
| ko01003.Glycosyltransferases. | 0.3685% | 0.3028% | 0.2809% | -0.2832 | 0.0164 | * | -0.1082 | 0.0184 | * |
| ko01004.Lipid.biosynthesis.proteins. | 0.6646% | 0.6475% | 0.6514% | -0.0376 | 0.9053 |  | 0.0087 | 0.9117 |  |
| ko01005.Lipopolysaccharide.biosynthesis.prote | 0.4336% | 0.3455% | 0.2934% | -0.3277 | 0.0282 | * | -0.2355 | 0.0277 | * |
| ko01006.Prenyltransferases. | 0.1415% | 0.1426% | 0.1392% | 0.0104 | 0.8632 |  | -0.0347 | 0.8588 |  |
| ko01007.Amino.acid.related.enzymes. | 1.7511% | 1.9556% | 1.9740% | 0.1594 | 0.0017 | * | 0.0135 | 0.0018 | * |
| ko01008.Polyketide.biosynthesis.proteins. | 0.0957% | 0.0450% | 0.0279% | -1.0870 | 0.0055 | * | -0.6911 | 0.0124 | * |
| ko01009.Protein.phosphatases.&.associated.proteins. | 0.1239% | 0.1569% | 0.1560% | 0.3412 | 0.0017 | * | -0.0084 | 0.0018 | * |
| ko01011.Peptidoglycan.biosynthesis.&.degradation.proteins. | 1.1544% | 1.2086% | 1.2632% | 0.0662 | 0.0017 | * | 0.0637 | 0.0018 | * |
| ko00536.Glycosaminoglycan.binding.proteins. | 0.0447% | 0.0374% | 0.0342% | -0.2581 | 0.8097 |  | -0.1295 | 0.8021 |  |
| ko00537.Glycosylphosphatidylinositol..GPI | 0.0742% | 0.0688% | 0.0695% | -0.1088 | 0.9359 |  | 0.0133 | 0.9323 |  |
| ko01504.Antimicrobial.resistance.genes. | 0.3563% | 0.2812% | 0.2605% | -0.3415 | 0.0105 | * | -0.1100 | 0.0102 | * |
| ko02000.Transporters. | 11.1944% | 9.9927% | 9.5069% | -0.1638 | 0.0017 | * | -0.0719 | 0.0018 | * |
| ko02022.Two.component.system. | 0.6974% | 0.5363% | 0.5626% | -0.3790 | 0.0390 | * | 0.0690 | 0.0387 | * |
| ko02035.Bacterial.motility.proteins. | 0.5028% | 0.3026% | 0.2898% | -0.7324 | 0.0153 | * | -0.0624 | 0.0066 | * |
| ko02042.Bacterial.toxins. | 0.1848% | 0.1828% | 0.1593% | -0.0161 | 0.7518 |  | -0.1987 | 0.7594 |  |
| ko02044.Secretion.system. | 1.3521% | 0.8866% | 0.8500% | -0.6088 | 0.0017 | * | -0.0608 | 0.0018 | * |
| ko02048.Prokaryotic.defense.system. | 0.8460% | 0.8667% | 0.9287% | 0.0350 | 0.1375 |  | 0.0995 | 0.1215 |  |
| ko04090.CD.molecules. | 0.0697% | 0.0746% | 0.0707% | 0.0983 | 0.9226 |  | -0.0779 | 0.9236 |  |
| ko04147.Exosome. | 1.7135% | 1.8888% | 1.8852% | 0.1405 | 0.0379 | * | -0.0027 | 0.0422 | * |
| ko04812.Cytoskeleton.proteins. | 0.2512% | 0.3221% | 0.3363% | 0.3584 | 0.0017 | * | 0.0622 | 0.0018 | * |
| ko04112.Cell.cycle...Caulobacter. | 0.3987% | 0.4769% | 0.4986% | 0.2583 | 0.0017 | * | 0.0640 | 0.0018 | * |
| ko04214.Apoptosis...fly. | 0.0237% | 0.0224% | 0.0216% | -0.0762 | 0.6831 |  | -0.0554 | 0.6656 |  |
| ko04216.Ferroptosis. | 0.1704% | 0.1732% | 0.1694% | 0.0228 | 0.9885 |  | -0.0321 | 0.9865 |  |
| ko04217.Necroptosis. | 0.2549% | 0.2984% | 0.3192% | 0.2274 | 0.0017 | * | 0.0972 | 0.0018 | * |
| ko02030.Bacterial.chemotaxis. | 0.1625% | 0.1188% | 0.1214% | -0.4520 | 0.3529 |  | 0.0319 | 0.3627 |  |
| ko02040.Flagellar.assembly. | 0.1867% | 0.1059% | 0.0929% | -0.8176 | 0.0616 |  | -0.1888 | 0.0592 | * |
| ko02024.Quorum.sensing. | 1.8498% | 1.8060% | 1.7838% | -0.0346 | 0.9133 |  | -0.0179 | 0.9117 |  |
| ko02025.Biofilm.formation...Pseudomonas. | 0.2547% | 0.1518% | 0.1396% | -0.7464 | 0.0017 | * | -0.1211 | 0.0018 | * |
| ko02026.Biofilm.formation...Escherichia.coli. | 0.5061% | 0.3459% | 0.3416% | -0.5489 | 0.0017 | * | -0.0182 | 0.0031 | * |
| ko05111.Biofilm.formation...Vibrio.cholerae. | 0.3273% | 0.2140% | 0.1738% | -0.6131 | 0.0017 | * | -0.3003 | 0.0018 | * |
| ko04138.Autophagy...yeast. | 0.0305% | 0.0300% | 0.0326% | -0.0212 | 0.9133 |  | 0.1161 | 0.9117 |  |
| ko04142.Lysosome. | 0.1718% | 0.2136% | 0.1900% | 0.3140 | 0.6016 |  | -0.1685 | 0.6283 |  |
| ko04146.Peroxisome. | 0.2546% | 0.2552% | 0.2483% | 0.0033 | 0.9638 |  | -0.0397 | 0.9658 |  |
| ko02010.ABC.transporters. | 2.7671% | 2.5299% | 2.5805% | -0.1293 | 0.2796 |  | 0.0286 | 0.2457 |  |
| ko02060.Phosphotransferase.system..PTS.. | 0.7202% | 0.5493% | 0.4870% | -0.3909 | 0.0264 | * | -0.1737 | 0.0278 | * |
| ko03070.Bacterial.secretion.system. | 0.6128% | 0.5389% | 0.5446% | -0.1855 | 0.1158 |  | 0.0154 | 0.1117 |  |
| ko02020.Two.component.system. | 1.7401% | 1.3607% | 1.3354% | -0.3549 | 0.0207 | * | -0.0270 | 0.0264 | * |
| ko04016.MAPK.signaling.pathway...plant. | 0.1084% | 0.0899% | 0.0867% | -0.2700 | 0.0368 | * | -0.0521 | 0.0417 | * |
| ko04066.HIF.1.signaling.pathway. | 0.1886% | 0.2280% | 0.2231% | 0.2742 | 0.0017 | * | -0.0319 | 0.0018 | * |
| ko04068.FoxO.signaling.pathway. | 0.0246% | 0.0181% | 0.0153% | -0.4433 | 0.0701 |  | -0.2437 | 0.0724 |  |
| ko04070.Phosphatidylinositol.signaling.system. | 0.0593% | 0.0569% | 0.0561% | -0.0581 | 0.7518 |  | -0.0214 | 0.7594 |  |
| ko04151.PI3K.Akt.signaling.pathway. | 0.0194% | 0.0336% | 0.0377% | 0.7881 | 0.0017 | * | 0.1659 | 0.0031 | * |
| ko04152.AMPK.signaling.pathway. | 0.0290% | 0.0328% | 0.0314% | 0.1774 | 0.8542 |  | -0.0658 | 0.8575 |  |
| ko03018.RNA.degradation. | 0.7165% | 0.7993% | 0.8315% | 0.1578 | 0.0017 | * | 0.0569 | 0.0018 | * |
| ko03050.Proteasome. | 0.0303% | 0.0276% | 0.0272% | -0.1384 | 0.9053 |  | -0.0179 | 0.8898 |  |
| ko03060.Protein.export. | 0.4146% | 0.4707% | 0.4769% | 0.1830 | 0.0017 | * | 0.0191 | 0.0018 | * |
| ko04122.Sulfur.relay.system. | 0.1249% | 0.1380% | 0.1584% | 0.1438 | 0.0523 |  | 0.1988 | 0.0568 | * |
| ko04141.Protein.processing.in.ER | 0.0374% | 0.0504% | 0.0494% | 0.4282 | 0.0032 | * | -0.0279 | 0.0066 | * |
| ko03030.DNA.replication. | 0.7552% | 0.8334% | 0.8610% | 0.1422 | 0.0257 | * | 0.0470 | 0.0278 | * |
| ko03410.Base.excision.repair. | 0.3547% | 0.3705% | 0.3779% | 0.0629 | 0.0792 |  | 0.0284 | 0.0528 | * |
| ko03420.Nucleotide.excision.repair. | 0.7288% | 0.8035% | 0.8298% | 0.1408 | 0.1355 |  | 0.0464 | 0.1188 |  |
| ko03430.Mismatch.repair. | 0.8607% | 0.9817% | 1.0198% | 0.1898 | 0.0017 | * | 0.0549 | 0.0018 | * |
| ko03440.Homologous.recombination. | 1.0163% | 1.1278% | 1.1747% | 0.1501 | 0.0044 | * | 0.0588 | 0.0031 | * |
| ko03020.RNA.polymerase. | 0.3370% | 0.3902% | 0.3919% | 0.2114 | 0.0017 | * | 0.0063 | 0.0018 | * |
| ko00970.Aminoacyl.tRNA.biosynthesis. | 1.3881% | 1.5944% | 1.6343% | 0.1999 | 0.0017 | * | 0.0357 | 0.0018 | * |
| ko03008.Ribosome.biogenesis.in.eukaryotes. | 0.0527% | 0.0499% | 0.0516% | -0.0813 | 0.9185 |  | 0.0508 | 0.9190 |  |
| ko03010.Ribosome. | 0.8666% | 0.9639% | 0.9816% | 0.1536 | 0.0249 | * | 0.0263 | 0.0278 | * |
| ko03013.RNA.transport. | 0.0246% | 0.0359% | 0.0424% | 0.5462 | 0.0189 | * | 0.2397 | 0.0277 | * |
| ko00220.Arginine.biosynthesis. | 0.5794% | 0.6291% | 0.6528% | 0.1186 | 0.0017 | * | 0.0535 | 0.0018 | * |
| ko00250.Alanine..aspartate.&.glutamate.metabolism. | 1.1533% | 1.2817% | 1.2924% | 0.1523 | 0.0017 | * | 0.0121 | 0.0018 | * |
| ko00260.Glycine..serine.&.threonine.metabolism | 0.9838% | 0.9752% | 0.9561% | -0.0127 | 0.4790 |  | -0.0285 | 0.5119 |  |
| ko00270.Cysteine.and.methionine.metabolism. | 1.1260% | 1.1864% | 1.1933% | 0.0754 | 0.0844 |  | 0.0084 | 0.0911 |  |
| ko00280.Valine..leucine.&.isoleucine.degradation. | 0.2825% | 0.2708% | 0.2385% | -0.0612 | 0.0032 | * | -0.1828 | 0.0018 | * |
| ko00290.Valine..leucine.&.isoleucine.biosynthesis. | 0.5832% | 0.6150% | 0.6309% | 0.0766 | 0.2542 |  | 0.0369 | 0.2531 |  |
| ko00300.Lysine.biosynthesis. | 0.5797% | 0.6521% | 0.6819% | 0.1698 | 0.0017 | * | 0.0644 | 0.0044 | * |
| ko00310.Lysine.degradation. | 0.2402% | 0.1689% | 0.1465% | -0.5079 | 0.0017 | * | -0.2061 | 0.0018 | * |
| ko00330.Arginine.and.proline.metabolism. | 0.3982% | 0.3571% | 0.3511% | -0.1573 | 0.0390 | * | -0.0243 | 0.0285 | * |
| ko00340.Histidine.metabolism. | 0.2647% | 0.3116% | 0.3057% | 0.2355 | 0.0044 | * | -0.0275 | 0.0056 | * |
| ko00350.Tyrosine.metabolism. | 0.2456% | 0.2180% | 0.2156% | -0.1719 | 0.0368 | * | -0.0160 | 0.0293 | * |
| ko00360.Phenylalanine.metabolism. | 0.1885% | 0.1689% | 0.1611% | -0.1589 | 0.3527 |  | -0.0681 | 0.3928 |  |
| ko00380.Tryptophan.metabolism. | 0.1841% | 0.1243% | 0.1070% | -0.5659 | 0.0017 | * | -0.2165 | 0.0018 | * |
| ko00400.Phenylalanine..tyrosine.&.tryptophan.biosynthesis. | 0.6095% | 0.6760% | 0.6806% | 0.1494 | 0.0095 | * | 0.0096 | 0.0066 | * |
| ko00261.Monobactam.biosynthesis. | 0.2549% | 0.2513% | 0.2505% | -0.0202 | 0.8410 |  | -0.0049 | 0.8426 |  |
| ko00311.Penicillin.and.cephalosporin.biosynthesis. | 0.0362% | 0.0346% | 0.0317% | -0.0648 | 0.8270 |  | -0.1269 | 0.8242 |  |
| ko00332.Carbapenem.biosynthesis. | 0.0783% | 0.0858% | 0.0861% | 0.1310 | 0.2057 |  | 0.0055 | 0.1949 |  |
| ko00333.Prodigiosin.biosynthesis. | 0.0583% | 0.0718% | 0.0745% | 0.3005 | 0.0055 | * | 0.0538 | 0.0031 | * |
| ko00401.Novobiocin.biosynthesis. | 0.1118% | 0.1261% | 0.1289% | 0.1739 | 0.0017 | * | 0.0324 | 0.0018 | * |
| ko00405.Phenazine.biosynthesis. | 0.0508% | 0.0549% | 0.0506% | 0.1140 | 0.3519 |  | -0.1173 | 0.3480 |  |
| ko00521.Streptomycin.biosynthesis. | 0.2931% | 0.3160% | 0.3285% | 0.1083 | 0.2676 |  | 0.0560 | 0.2878 |  |
| ko00524.Neomycin..kanamycin.&.gentamicin.biosynthesis. | 0.0500% | 0.0553% | 0.0512% | 0.1459 | 0.8017 |  | -0.1125 | 0.7971 |  |
| ko00525.Acarbose.and.validamycin.biosynthesis | 0.0975% | 0.0965% | 0.0989% | -0.0140 | 0.9595 |  | 0.0355 | 0.9658 |  |
| ko00940.Phenylpropanoid.biosynthesis. | 0.2486% | 0.2804% | 0.3114% | 0.1740 | 0.2057 |  | 0.1512 | 0.2096 |  |
| ko00950.Isoquinoline.alkaloid.biosynthesis. | 0.0559% | 0.0589% | 0.0579% | 0.0737 | 0.7518 |  | -0.0235 | 0.7589 |  |
| ko00960.Tropane..piperidine.and.pyridine.alkaloid.biosynthesis. | 0.1169% | 0.1072% | 0.1026% | -0.1251 | 0.0249 | * | -0.0638 | 0.0327 | * |
| ko00966.Glucosinolate.biosynthesis. | 0.0475% | 0.0599% | 0.0642% | 0.3349 | 0.0032 | * | 0.0988 | 0.0018 | * |
| ko00010.Glycolysis...Gluconeogenesis. | 1.3685% | 1.3559% | 1.3429% | -0.0134 | 0.9006 |  | -0.0138 | 0.9029 |  |
| ko00020.Citrate.cycle..TCA.cycle.. | 0.6374% | 0.6633% | 0.6194% | 0.0573 | 0.6039 |  | -0.0986 | 0.5742 |  |
| ko00030.Pentose.phosphate.pathway. | 0.8751% | 0.9129% | 0.9080% | 0.0610 | 0.0903 |  | -0.0077 | 0.0827 |  |
| ko00040.Pentose.&.glucuronate.interconversions. | 0.4507% | 0.4659% | 0.4761% | 0.0480 | 0.8333 |  | 0.0312 | 0.8360 |  |
| ko00051.Fructose.and.mannose.metabolism. | 0.6741% | 0.7091% | 0.6586% | 0.0729 | 0.8400 |  | -0.1065 | 0.8242 |  |
| ko00052.Galactose.metabolism. | 1.3713% | 1.4104% | 1.4438% | 0.0405 | 0.6714 |  | 0.0338 | 0.6656 |  |
| ko00053.Ascorbate.and.aldarate.metabolism. | 0.2008% | 0.1606% | 0.1417% | -0.3225 | 0.0055 | * | -0.1809 | 0.0018 | * |
| ko00500.Starch.and.sucrose.metabolism. | 1.8831% | 1.7369% | 1.8598% | -0.1166 | 0.2676 |  | 0.0987 | 0.2759 |  |
| ko00520.Amino.sugar.&.nucleotide.sugar.metabolism. | 1.4274% | 1.5109% | 1.5059% | 0.0821 | 0.1237 |  | -0.0048 | 0.1215 |  |
| ko00562.Inositol.phosphate.metabolism. | 0.0927% | 0.0964% | 0.0929% | 0.0571 | 0.9414 |  | -0.0538 | 0.9460 |  |
| ko00620.Pyruvate.metabolism. | 1.1956% | 1.2027% | 1.1813% | 0.0085 | 0.9133 |  | -0.0259 | 0.9117 |  |
| ko00630.Glyoxylate.&.dicarboxylate.metabolism. | 0.8051% | 0.7903% | 0.7518% | -0.0269 | 0.3883 |  | -0.0721 | 0.3947 |  |
| ko00640.Propanoate.metabolism. | 0.7616% | 0.7048% | 0.6660% | -0.1119 | 0.0172 | * | -0.0817 | 0.0366 | * |
| ko00650.Butanoate.metabolism. | 0.6755% | 0.6790% | 0.6623% | 0.0075 | 0.9053 |  | -0.0360 | 0.9117 |  |
| ko00660.C5.Branched.dibasic.acid.metabolism. | 0.3002% | 0.3091% | 0.3104% | 0.0422 | 0.7518 |  | 0.0060 | 0.7594 |  |
| ko00190.Oxidative.phosphorylation. | 0.6758% | 0.7777% | 0.7946% | 0.2027 | 0.0032 | * | 0.0309 | 0.0044 | * |
| ko00195.Photosynthesis. | 0.2479% | 0.3078% | 0.3369% | 0.3123 | 0.0017 | * | 0.1304 | 0.0018 | * |
| ko00680.Methane.metabolism. | 0.7654% | 0.7906% | 0.7986% | 0.0467 | 0.0746 |  | 0.0146 | 0.0703 |  |
| ko00710.Carbon.fixation.in.photosynthetic. | 0.5582% | 0.5915% | 0.5875% | 0.0836 | 0.0310 | * | -0.0099 | 0.0221 | * |
| ko00720.Carbon.fixation.pathways.prokkaryotes. | 0.8798% | 0.9867% | 0.9634% | 0.1655 | 0.0117 | * | -0.0344 | 0.0056 | * |
| ko00910.Nitrogen.metabolism. | 0.5499% | 0.5312% | 0.5179% | -0.0500 | 0.4417 |  | -0.0366 | 0.4577 |  |
| ko00920.Sulfur.metabolism. | 0.2802% | 0.2369% | 0.2392% | -0.2419 | 0.3382 |  | 0.0135 | 0.3547 |  |
| ko00511.Other.glycan.degradation. | 0.6352% | 0.7521% | 0.6812% | 0.2436 | 0.5678 |  | -0.1428 | 0.5591 |  |
| ko00513.Various.types.of.N.glycan.biosynthesis. | 0.0626% | 0.0912% | 0.0783% | 0.5425 | 0.6016 |  | -0.2206 | 0.5793 |  |
| ko00531.Glycosaminoglycan.degradation. | 0.1366% | 0.1785% | 0.1637% | 0.3857 | 0.5678 |  | -0.1245 | 0.5591 |  |
| ko00540.Lipopolysaccharide.biosynthesis. | 0.2663% | 0.1835% | 0.1367% | -0.5372 | 0.0216 | * | -0.4251 | 0.0330 | * |
| ko00550.Peptidoglycan.biosynthesis. | 0.7277% | 0.8375% | 0.8960% | 0.2029 | 0.0017 | * | 0.0973 | 0.0018 | * |
| ko00572.Arabinogalactan.biosynthesis...Mycobacterium. | 0.0405% | 0.0426% | 0.0352% | 0.0706 | 0.6494 |  | -0.2725 | 0.6392 |  |
| ko00603.Glycosphingolipid.biosynthesis..globo.&.isoglobo.series | 0.1730% | 0.2173% | 0.2123% | 0.3288 | 0.2676 |  | -0.0330 | 0.2531 |  |
| ko00604.Glycosphingolipid.biosynthesis...ganglio.series. | 0.0626% | 0.0912% | 0.0782% | 0.5419 | 0.5857 |  | -0.2210 | 0.5742 |  |
| ko00061.Fatty.acid.biosynthesis. | 0.5617% | 0.6109% | 0.6386% | 0.1212 | 0.3423 |  | 0.0640 | 0.3204 |  |
| ko00071.Fatty.acid.degradation. | 0.4147% | 0.3434% | 0.3355% | -0.2724 | 0.0207 | * | -0.0334 | 0.0189 | * |
| ko00072.Synthesis.&.degradation.of.ketone.bodies. | 0.0243% | 0.0187% | 0.0188% | -0.3762 | 0.4417 |  | 0.0074 | 0.4577 |  |
| ko00120.Primary.bile.acid.biosynthesis. | 0.0296% | 0.0389% | 0.0367% | 0.3947 | 0.0861 |  | -0.0845 | 0.0828 |  |
| ko00121.Secondary.bile.acid.biosynthesis. | 0.0451% | 0.0612% | 0.0670% | 0.4430 | 0.0017 | * | 0.1299 | 0.0018 | * |
| ko00561.Glycerolipid.metabolism. | 0.3269% | 0.3346% | 0.3452% | 0.0339 | 0.7449 |  | 0.0449 | 0.7517 |  |
| ko00564.Glycerophospholipid.metabolism. | 0.3457% | 0.3173% | 0.3299% | -0.1238 | 0.5306 |  | 0.0563 | 0.5204 |  |
| ko00600.Sphingolipid.metabolism. | 0.4365% | 0.5085% | 0.5060% | 0.2203 | 0.3423 |  | -0.0071 | 0.3671 |  |
| ko01040.Biosynthesis.of.unsaturated.fatty.acids. | 0.0311% | 0.0209% | 0.0204% | -0.5722 | 0.0044 | * | -0.0361 | 0.0044 | * |
| ko00130.Ubiquinone.&.other.terpenoid.quinone.biosynthesis. | 0.1853% | 0.1373% | 0.1041% | -0.4324 | 0.0017 | * | -0.4003 | 0.0018 | * |
| ko00670.One.carbon.pool.by.folate. | 0.3954% | 0.4410% | 0.4408% | 0.1574 | 0.0017 | * | -0.0006 | 0.0018 | * |
| ko00730.Thiamine.metabolism. | 0.4217% | 0.4692% | 0.4917% | 0.1540 | 0.0017 | * | 0.0676 | 0.0018 | * |
| ko00740.Riboflavin.metabolism. | 0.1298% | 0.1289% | 0.1193% | -0.0102 | 0.6152 |  | -0.1115 | 0.6176 |  |
| ko00750.Vitamin.B6.metabolism. | 0.1848% | 0.1876% | 0.1798% | 0.0221 | 0.6494 |  | -0.0619 | 0.6283 |  |
| ko00760.Nicotinate.&.nicotinamide.metabolism. | 0.4937% | 0.4886% | 0.4749% | -0.0151 | 0.8410 |  | -0.0410 | 0.8242 |  |
| ko00770.Pantothenate.and.CoA.biosynthesis. | 0.4835% | 0.5153% | 0.5281% | 0.0918 | 0.0017 | * | 0.0355 | 0.0018 | * |
| ko00780.Biotin.metabolism. | 0.2026% | 0.2197% | 0.2165% | 0.1169 | 0.8042 |  | -0.0211 | 0.7840 |  |
| ko00785.Lipoic.acid.metabolism. | 0.0464% | 0.0389% | 0.0340% | -0.2530 | 0.0055 | * | -0.1966 | 0.0031 | * |
| ko00790.Folate.biosynthesis. | 0.3449% | 0.3550% | 0.3434% | 0.0414 | 0.7518 |  | -0.0479 | 0.7594 |  |
| ko00830.Retinol.metabolism. | 0.0380% | 0.0278% | 0.0241% | -0.4503 | 0.0017 | * | -0.2066 | 0.0018 | * |
| ko00860.Porphyrin.and.chlorophyll.metabolism. | 0.3608% | 0.4686% | 0.4772% | 0.3773 | 0.0350 | * | 0.0261 | 0.0272 | * |
| ko00410.beta.Alanine.metabolism. | 0.1435% | 0.1110% | 0.0981% | -0.3713 | 0.0244 | * | -0.1776 | 0.0189 | * |
| ko00430.Taurine.and.hypotaurine.metabolism. | 0.1377% | 0.1276% | 0.1256% | -0.1100 | 0.0179 | * | -0.0232 | 0.0189 | * |
| ko00440.Phosphonate.&.phosphinate.metabolism. | 0.0735% | 0.0510% | 0.0497% | -0.5266 | 0.0313 | * | -0.0374 | 0.0310 | * |
| ko00450.Selenocompound.metabolism. | 0.5215% | 0.4947% | 0.4888% | -0.0761 | 0.0455 | * | -0.0171 | 0.0571 | * |
| ko00460.Cyanoamino.acid.metabolism. | 0.3234% | 0.3647% | 0.4027% | 0.1735 | 0.0748 |  | 0.1428 | 0.0666 |  |
| ko00471.D.Glutamine.&.D.glutamate.metabolism | 0.1277% | 0.1505% | 0.1519% | 0.2376 | 0.0017 | * | 0.0132 | 0.0018 | * |
| ko00473.D.Alanine.metabolism. | 0.0979% | 0.1084% | 0.1113% | 0.1470 | 0.0044 | * | 0.0390 | 0.0031 | * |
| ko00480.Glutathione.metabolism. | 0.3967% | 0.3222% | 0.2898% | -0.3002 | 0.0017 | * | -0.1532 | 0.0018 | * |
| ko00281.Geraniol.degradation. | 0.0384% | 0.0120% | 0.0087% | -1.6757 | 0.0017 | * | -0.4597 | 0.0018 | * |
| ko00523.Polyketide.sugar.unit.biosynthesis. | 0.1459% | 0.1547% | 0.1613% | 0.0846 | 0.6526 |  | 0.0606 | 0.6656 |  |
| ko00900.Terpenoid.backbone.biosynthesis. | 0.3429% | 0.3828% | 0.3967% | 0.1588 | 0.0017 | * | 0.0514 | 0.0018 | * |
| ko00903.Limonene.and.pinene.degradation. | 0.0492% | 0.0344% | 0.0291% | -0.5157 | 0.0017 | * | -0.2400 | 0.0018 | * |
| ko00908.Zeatin.biosynthesis. | 0.0284% | 0.0322% | 0.0324% | 0.1831 | 0.0451 | * | 0.0092 | 0.0483 | * |
| ko00981.Insect.hormone.biosynthesis. | 0.0265% | 0.0272% | 0.0239% | 0.0347 | 0.8333 |  | -0.1870 | 0.8242 |  |
| ko01051.Biosynthesis.of.ansamycins. | 0.1148% | 0.0991% | 0.0944% | -0.2122 | 0.0356 | * | -0.0698 | 0.0387 | * |
| ko01053.Biosynthesis.of.siderophore.group.nr.peptides | 0.1214% | 0.0541% | 0.0368% | -1.1651 | 0.0017 | * | -0.5557 | 0.0044 | * |
| ko01055.Biosynthesis.of.vancomycin.group.antibiotics. | 0.0551% | 0.0513% | 0.0536% | -0.1026 | 0.8632 |  | 0.0614 | 0.8588 |  |
| ko09113.Global.maps.only. | 0.0451% | 0.0411% | 0.0453% | -0.1360 | 0.8090 |  | 0.1428 | 0.7985 |  |
| ko00230.Purine.metabolism. | 1.7736% | 1.8561% | 1.8568% | 0.0656 | 0.0179 | * | 0.0005 | 0.0078 | * |
| ko00240.Pyrimidine.metabolism. | 1.0619% | 1.1472% | 1.1708% | 0.1115 | 0.0017 | * | 0.0293 | 0.0018 | * |
| ko00362.Benzoate.degradation. | 0.1000% | 0.0619% | 0.0617% | -0.6907 | 0.0313 | * | -0.0064 | 0.0417 | * |
| ko00625.Chloroaklane.&.chloroalkene.degradation. | 0.1701% | 0.1406% | 0.1451% | -0.2742 | 0.0663 |  | 0.0447 | 0.0713 |  |
| ko00626.Naphthalene.degradation. | 0.1315% | 0.1067% | 0.1096% | -0.3014 | 0.0379 | * | 0.0396 | 0.0528 | * |
| ko00627.Aminobenzoate.degradation. | 0.0366% | 0.0230% | 0.0208% | -0.6700 | 0.0017 | * | -0.1430 | 0.0056 | * |
| ko00633.Nitrotoluene.degradation. | 0.0561% | 0.0494% | 0.0365% | -0.1833 | 0.1998 |  | -0.4376 | 0.1949 |  |
| ko00643.Styrene.degradation. | 0.0153% | 0.0115% | 0.0128% | -0.4129 | 0.7083 |  | 0.1584 | 0.7381 |  |
| ko00791.Atrazine.degradation. | 0.0156% | 0.0165% | 0.0132% | 0.0842 | 0.9053 |  | -0.3194 | 0.9117 |  |
| ko00930.Caprolactam.degradation. | 0.0235% | 0.0090% | 0.0078% | -1.3750 | 0.0017 | * | -0.2208 | 0.0031 | * |
| ko00980.Metabolism.of.xenobiotics.by.cytochrome.P450. | 0.0507% | 0.0308% | 0.0261% | -0.7179 | 0.0017 | * | -0.2415 | 0.0018 | * |
| ko00982.Drug.metabolism...cytochrome.P450. | 0.0507% | 0.0308% | 0.0260% | -0.7191 | 0.0017 | * | -0.2458 | 0.0018 | * |
| ko00983.Drug.metabolism...other.enzymes. | 0.2675% | 0.2754% | 0.2785% | 0.0420 | 0.4790 |  | 0.0165 | 0.4577 |  |

Table 3*. Lipid metabolites included in analyses with annotations, median mass (m/z), median retention time in seconds (rt), and valence.*

| Lipid | Median m/z | Median rt (seconds) | Valence |
| --- | --- | --- | --- |
| So(d18:1)+H | 300.2893 | 90.6687 | ve+ |
| So(d18:0)+H | 302.3047 | 104.1785 | ve+ |
| MG(18:2)+H | 355.2839 | 65.4578 | ve+ |
| MG 17:2; [M+NH4]+ | 358.2948 | 40.9098 | ve+ |
| MG 17:2; [M+NH4]+ | 358.2950 | 56.4968 | ve+ |
| MG 18:3; [M+NH4]+ | 370.2949 | 199.5786 | ve+ |
| MG 18:3; [M+NH4]+ | 370.2944 | 42.2456 | ve+ |
| MG 18:3; [M+NH4]+ | 370.2945 | 59.0423 | ve+ |
| MG 18:1; [M+NH4]+ | 374.3262 | 208.2640 | ve+ |
| So(d24:0)+H | 386.3994 | 262.3042 | ve+ |
| MG 20:5; [M+NH4]+ | 394.2944 | 179.8314 | ve+ |
| MG 20:5; [M+NH4]+ | 394.3010 | 225.9909 | ve+ |
| MG 20:2; [M+NH4]+ | 400.3411 | 126.5315 | ve+ |
| MG 20:2; [M+NH4]+ | 400.3422 | 213.6014 | ve+ |
| MG 20:1; [M+NH4]+ | 402.3561 | 127.4914 | ve+ |
| DG(8:0/12:0)+Na | 423.3073 | 231.1439 | ve+ |
| MG 22:4; [M+NH4]+ | 424.3415 | 101.1744 | ve+ |
| MG 22:2; [M+NH4]+ | 428.3726 | 189.1778 | ve+ |
| DG 21:0; [M+NH4]+ | 432.3684 | 215.6697 | ve+ |
| DG(10:0/12:0)+Na | 451.3393 | 271.6578 | ve+ |
| PE(16:0e)+H | 454.2918 | 110.4404 | ve+ |
| PE(16:0e)+H | 454.2918 | 404.7121 | ve+ |
| DG(12:0/12:0)+Na | 479.3704 | 315.0194 | ve+ |
| PE(18:0e)+H | 482.3238 | 176.8222 | ve+ |
| MG(27:1)+H | 483.4402 | 398.2040 | ve+ |
| MG 26:2; [M+NH4]+ | 484.4437 | 398.2227 | ve+ |
| LPC(16:0)+H | 496.3393 | 108.7744 | ve+ |
| LPC(16:0)+H | 496.3392 | 77.7938 | ve+ |
| LPC(18:0e)+H | 510.3917 | 200.4979 | ve+ |
| Cer(d16:1/16:0)+H | 510.4872 | 375.1542 | ve+ |
| Cer(d16:0/16:0)+H | 512.5031 | 393.0604 | ve+ |
| LPC(16:0)+Na | 518.3202 | 110.4280 | ve+ |
| LPC(18:2)+H | 520.3391 | 86.8546 | ve+ |
| lysoPC 18:1; [M+H]+ | 522.3543 | 121.7223 | ve+ |
| LPC(18:0)+H | 524.3706 | 174.9517 | ve+ |
| LPC(18:0)+H | 524.3713 | 201.8440 | ve+ |
| Cer(d17:1/16:0)+H | 524.5035 | 401.5223 | ve+ |
| DG 29:5; [M+NH4]+ | 534.4193 | 367.5207 | ve+ |
| Cer(d18:2/16:0)+H | 536.5032 | 382.6523 | ve+ |
| Cer(d16:0/18:2)+H | 536.5025 | 405.4259 | ve+ |
| Cer(d18:1/16:0)+H | 538.5180 | 426.6776 | ve+ |
| Cer(d16:0/18:0)+H | 540.5342 | 384.1472 | ve+ |
| TG(8:0/8:0/12:0)+NH4 | 544.4560 | 378.0697 | ve+ |
| LPC(18:0)+Na | 546.3522 | 176.9351 | ve+ |
| Cer(d18:1/16:0+O)+H | 554.5133 | 405.3322 | ve+ |
| Cer(d18:1/18:2)+H | 562.5186 | 393.2536 | ve+ |
| Cer(d34:0)+Na | 562.5159 | 444.3866 | ve+ |
| Cer(d18:1/18:1)+H | 564.5333 | 433.0468 | ve+ |
| Cer(d18:0/18:0)+H | 568.5651 | 495.1868 | ve+ |
| Cer(d18:0/17:0+O)+H | 570.5444 | 406.7762 | ve+ |
| TG(8:0/10:0/12:0)+NH4 | 572.4876 | 425.0792 | ve+ |
| DG 31:0; [M+NH4]+ | 572.5238 | 381.3417 | ve+ |
| TG(8:0/10:0/12:0)+Na | 577.4418 | 424.7958 | ve+ |
| Cer(d18:1/18:1+O)+H | 580.5290 | 368.1170 | ve+ |
| TG(33:0p)+H | 581.5132 | 343.5467 | ve+ |
| DG 32:1; [M+NH4]+ | 584.5314 | 372.7194 | ve+ |
| Cer(d18:0/18:2)+Na | 586.5187 | 409.1560 | ve+ |
| Cer(d18:1/20:3)+H | 588.5338 | 406.5859 | ve+ |
| Cer(d36:1)+Na | 588.5344 | 447.8219 | ve+ |
| MG(34:4)+NH4 | 592.5293 | 471.9896 | ve+ |
| TG(8:0/12:0/12:0)+NH4 | 600.5193 | 472.5558 | ve+ |
| DG 34:4; [M+NH4]+ | 606.5139 | 372.7392 | ve+ |
| Cer(d18:1/20:0+O)+H | 610.5762 | 497.3957 | ve+ |
| DG(18:2/18:2)+H | 617.5128 | 241.2975 | ve+ |
| TG(18:0/8:0/8:0)+NH4 | 628.5499 | 472.4217 | ve+ |
| DG(18:3/18:2)+NH4 | 632.5228 | 405.5564 | ve+ |
| DG(16:0/20:4)+Na | 639.4936 | 439.3621 | ve+ |
| DG 37:7; [M+NH4]+ | 642.5120 | 359.5073 | ve+ |
| TG(6:0/12:0/18:0)+NH4 | 656.5815 | 518.3783 | ve+ |
| DG 38:6; [M+NH4]+ | 658.5351 | 359.5893 | ve+ |
| SM(d32:1)+H | 675.5427 | 320.6272 | ve+ |
| SM 33:0; [M]+ | 691.5678 | 368.6894 | ve+ |
| CerG1(d18:1/16:0)+H | 700.5714 | 384.4641 | ve+ |
| CerG1(d18:0/16:0)+H | 702.5857 | 403.8713 | ve+ |
| SM(d34:1)+H | 703.5725 | 369.8539 | ve+ |
| PE(16:0/18:2)+H | 716.5224 | 321.4063 | ve+ |
| PE(16:0/18:1)+H | 718.5380 | 363.7540 | ve+ |
| CerG1(d18:0/16:0+O)+H | 718.5818 | 390.0669 | ve+ |
| CerG1(d36:1)+H | 728.6025 | 434.4186 | ve+ |
| SM(d36:1)+H | 731.6033 | 426.9389 | ve+ |
| CerG1(d34:1+O)+Na | 738.5473 | 368.9983 | ve+ |
| PE 34:1; [M+Na]+ | 740.5218 | 297.1750 | ve+ |
| PC(16:0/18:1)+H | 760.5833 | 432.6865 | ve+ |
| CerG2(d18:1/16:0+O)+H | 878.6188 | 356.9982 | ve+ |
| FA(20:4)-H | 303.2332 | 194.6611 | ve- |
| FA(22:5)-H | 329.2490 | 201.2894 | ve- |
| cPA(18:0)-H | 419.2575 | 190.5491 | ve- |
| LPMe(18:0)-H | 451.2830 | 199.6275 | ve- |
| LPE(16:0)-H | 452.2783 | 110.5347 | ve- |
| LPE(18:0p)-H | 464.3154 | 195.6803 | ve- |
| LPE(18:0)-H | 480.3101 | 176.7309 | ve- |
| LPE(20:0p)-H | 492.3473 | 240.9356 | ve- |
| MGMG(16:0)+HCOO | 537.3275 | 126.9163 | ve- |
| OAHFA(18:2/18:1)-H | 559.4727 | 422.4298 | ve- |
| OAHFA(18:2/18:0)-H | 561.4895 | 459.6248 | ve- |
| OAHFA(18:1/18:0)-H | 563.5046 | 494.5790 | ve- |
| LPC(18:0)+HCOO | 568.3628 | 174.5876 | ve- |
| PE 25:0; [M-H]- | 592.3958 | 254.5163 | ve- |
| PE 27:0; [M-H]- | 620.4257 | 263.8740 | ve- |
| PE 27:0; [M-H]- | 620.4255 | 283.6201 | ve- |
| PG 27:1; [M-H]- | 649.4128 | 231.5340 | ve- |
| PG 27:0; [M-H]- | 651.4233 | 224.6613 | ve- |
| PE 34:2; [M-H]- | 714.5089 | 323.3438 | ve- |
| OAHFA(18:1/31:0)-H | 745.7051 | 430.4391 | ve- |
| PE 36:0; [M-H]- | 746.5794 | 401.1478 | ve- |
| PG(16:0/18:1)-H | 747.5179 | 366.3311 | ve- |
| SM(d18:1/16:0)+HCOO | 747.5659 | 370.7628 | ve- |
| MGDG 34:3; [M-H]- | 751.5315 | 368.1728 | ve- |
| PE 38:1; [M-H]- | 772.5931 | 432.5271 | ve- |
| MGDG 36:3; [M-H]- | 779.5731 | 349.4803 | ve- |
| PG 38:5; [M-H]- | 795.5131 | 313.1455 | ve- |
| PG 38:4; [M-H]- | 797.5301 | 328.7574 | ve- |
| MGDG(16:0/18:2)+HCOO | 799.5589 | 416.4379 | ve- |
| PE 40:1; [M-H]- | 800.6264 | 480.9434 | ve- |
| MGDG(16:0/18:1)+HCOO | 801.5747 | 454.2862 | ve- |
| PG 39:4; [M-H]- | 811.5530 | 314.3345 | ve- |
| MGDG(16:0/19:1)+HCOO | 815.5902 | 486.7226 | ve- |
| PG 40:4; [M-H]- | 825.5707 | 329.8479 | ve- |
| PG 40:3; [M-H]- | 827.5889 | 490.2816 | ve- |
| MGDG(16:0/20:1)+HCOO | 829.6065 | 498.7475 | ve- |
| PE 43:0; [M-H]- | 844.6852 | 540.5393 | ve- |
| PE 44:1; [M-H]- | 856.6889 | 567.7303 | ve- |
| DGDG(16:0/18:1)+HCOO | 963.6276 | 419.0568 | ve- |
|  |  |  |  |

Table 4*. HILIC (aqueous) metabolites included in analyses with annotations, median mass (m/z), median retention time in seconds (rt), and valence.*

| HILIC (aqueous) | Median m/z | Median rt (seconds) | Valence |
| --- | --- | --- | --- |
| M61T446 | 60.0401 | 445.6472 | ve+ |
| M61T447 | 61.0401 | 446.6472 | ve+ |
| M172T447 | 172.0606 | 447.1242 | ve+ |
| M191T447 | 191.0744 | 447.1286 | ve+ |
| M190T447 | 190.0707 | 447.1352 | ve+ |
| M130T447 | 130.0503 | 447.1436 | ve+ |
| M133T453 | 133.0611 | 453.1684 | ve+ |
| M244T466 | 243.9900 | 466.4442 | ve+ |
| M247T490 | 247.1436 | 490.0481 | ve+ |
| M159T499 | 159.0278 | 499.4561 | ve+ |
| M137T500 | 137.0459 | 499.9109 | ve+ |
| M138T500 | 138.0489 | 500.4332 | ve+ |
| M166T502 | 166.0724 | 501.9333 | ve+ |
| M189T516 | 189.0868 | 515.8054 | ve+ |
| M136T520 | 136.0619 | 520.4282 | ve+ |
| M153T521 | 153.0409 | 521.0790 | ve+ |
| M166T525 | 166.0723 | 524.8556 | ve+ |
| M144T527 | 144.1018 | 526.9302 | ve+ |
| M182T548 | 182.0673 | 548.2498 | ve+ |
| M118T557 | 118.0866 | 557.1974 | ve+ |
| M269T579 | 269.1219 | 578.5630 | ve+ |
| M146T579 | 146.1176 | 579.0733 | ve+ |
| M292T589 | 292.1742 | 589.2366 | ve+ |
| M246T593 | 246.1088 | 592.5101 | ve+ |
| M138T599 | 138.0551 | 599.3409 | ve+ |
| M166T600 | 166.0865 | 599.6542 | ve+ |
| M167T600 | 167.0897 | 599.8028 | ve+ |
| M120T600 | 120.0811 | 599.8054 | ve+ |
| M143T603 | 143.1682 | 603.2322 | ve+ |
| M142T603 | 142.1646 | 603.3100 | ve+ |
| M96T603 | 96.1596 | 603.3100 | ve+ |
| M217T614 | 217.1549 | 613.9880 | ve+ |
| M256T615 | 256.1035 | 615.0550 | ve+ |
| M105T615 | 105.1107 | 615.4220 | ve+ |
| M222T624 | 222.0967 | 623.9120 | ve+ |
| M265T624 | 265.1510 | 624.0750 | ve+ |
| M244T624 | 244.0793 | 624.2500 | ve+ |
| M204T624 | 204.0865 | 624.3700 | ve+ |
| M207T626 | 207.1493 | 625.5320 | ve+ |
| M173T627 | 173.1650 | 626.9420 | ve+ |
| M465T627 | 465.1674 | 627.0760 | ve+ |
| M433T629 | 433.2352 | 629.2420 | ve+ |
| M246T631 | 246.1087 | 630.5780 | ve+ |
| M162T637 | 162.0764 | 637.4160 | ve+ |
| M188T639 | 188.0707 | 639.2780 | ve+ |
| M210T640 | 210.1283 | 639.6720 | ve+ |
| M187T640 | 187.1553 | 640.2030 | ve+ |
| M192T640 | 192.0954 | 640.4160 | ve+ |
| M209T641 | 209.1226 | 640.5400 | ve+ |
| M162T641 | 162.1127 | 640.7910 | ve+ |
| M168T641 | 168.1129 | 640.9920 | ve+ |
| M183T641 | 182.6275 | 640.9990 | ve+ |
| M163T641 | 163.1156 | 641.0040 | ve+ |
| M252T641 | 252.1426 | 641.4430 | ve+ |
| M159T644 | 159.1491 | 643.7080 | ve+ |
| M160T645 | 160.1526 | 645.1580 | ve+ |
| M133T645 | 133.0319 | 645.2240 | ve+ |
| M72T646 | 72.0813 | 645.6960 | ve+ |
| M231T648 | 231.1899 | 647.9760 | ve+ |
| M230T648 | 230.1866 | 648.2120 | ve+ |
| M233T651 | 233.1496 | 650.6620 | ve+ |
| M407T651 | 407.2196 | 650.7150 | ve+ |
| M130T652 | 130.0867 | 652.1720 | ve+ |
| M123T655 | 123.0557 | 654.9960 | ve+ |
| M141T656 | 141.0658 | 655.9780 | ve+ |
| M116T659 | 116.0711 | 658.9960 | ve+ |
| M117T659 | 117.0743 | 659.0380 | ve+ |
| M139T659 | 139.0945 | 659.0770 | ve+ |
| M154T659 | 154.0976 | 659.4440 | ve+ |
| M70T659 | 70.0656 | 659.4480 | ve+ |
| M136T659 | 136.0868 | 659.4780 | ve+ |
| M121T660 | 121.0650 | 659.5400 | ve+ |
| M146T660 | 146.0812 | 660.2020 | ve+ |
| M155T661 | 155.1009 | 661.2800 | ve+ |
| M434T662 | 434.1901 | 661.9440 | ve+ |
| M435T662 | 435.1935 | 661.9860 | ve+ |
| M173T662 | 173.1395 | 662.3900 | ve+ |
| M128T663 | 128.1072 | 662.7570 | ve+ |
| M146T663 | 146.1368 | 663.4260 | ve+ |
| M145T663 | 145.1337 | 663.4820 | ve+ |
| M146T664 | 146.1300 | 663.9570 | ve+ |
| M174T664 | 174.1428 | 664.0740 | ve+ |
| M72T667 | 72.0813 | 666.6670 | ve+ |
| M254T668 | 254.1606 | 668.1050 | ve+ |
| M277T669 | 277.1761 | 669.3140 | ve+ |
| M100T674 | 100.0761 | 674.0260 | ve+ |
| M164T675 | 164.1284 | 675.0280 | ve+ |
| M137T677 | 137.0707 | 677.1310 | ve+ |
| M133T678 | 133.1215 | 678.2640 | ve+ |
| M114T679 | 114.0917 | 678.8000 | ve+ |
| M132T679 | 132.1216 | 679.2880 | ve+ |
| M150T682 | 150.1127 | 682.3760 | ve+ |
| M265T685 | 265.1109 | 685.3420 | ve+ |
| M273T688 | 273.2538 | 687.7350 | ve+ |
| M76T689 | 76.0762 | 688.8040 | ve+ |
| M204T692 | 204.0867 | 691.6350 | ve+ |
| M165T704 | 165.0548 | 703.5780 | ve+ |
| M182T704 | 182.0810 | 703.6640 | ve+ |
| M105T722 | 105.0743 | 721.5170 | ve+ |
| M86T723 | 86.0606 | 722.5840 | ve+ |
| M279T728 | 279.1014 | 728.2640 | ve+ |
| M407T728 | 407.1363 | 728.3520 | ve+ |
| M175T741 | 175.1081 | 740.7540 | ve+ |
| M252T758 | 252.1444 | 758.2380 | ve+ |
| M217T760 | 217.1295 | 759.9180 | ve+ |
| M90T766 | 90.0554 | 765.8310 | ve+ |
| M91T766 | 91.0587 | 765.9480 | ve+ |
| M265T772 | 265.1223 | 771.7480 | ve+ |
| M264T772 | 264.1180 | 772.2400 | ve+ |
| M258T778 | 258.1104 | 777.9050 | ve+ |
| M120T780 | 120.0659 | 779.9040 | ve+ |
| M126T793 | 126.0223 | 792.8580 | ve+ |
| M149T805 | 149.0638 | 804.8040 | ve+ |
| M148T805 | 148.0606 | 804.8050 | ve+ |
| M84T806 | 84.0448 | 805.5880 | ve+ |
| M265T806 | 265.1222 | 805.8160 | ve+ |
| M130T806 | 130.0503 | 806.0100 | ve+ |
| M264T806 | 264.1186 | 806.2720 | ve+ |
| M147T808 | 147.0763 | 807.7950 | ve+ |
| M76T809 | 76.0398 | 809.1060 | ve+ |
| M161T810 | 161.0920 | 809.5500 | ve+ |
| M292T815 | 292.1032 | 815.1460 | ve+ |
| M311T815 | 311.1173 | 815.2720 | ve+ |
| M310T815 | 310.1120 | 815.2870 | ve+ |
| M351T824 | 351.1582 | 823.8970 | ve+ |
| M106T832 | 106.0503 | 832.2120 | ve+ |
| M134T847 | 134.0450 | 846.6520 | ve+ |
| M88T847 | 88.0398 | 846.6560 | ve+ |
| M116T847 | 116.0345 | 847.1500 | ve+ |
| M266T858 | 266.1589 | 858.3200 | ve+ |
| M454T860 | 454.1559 | 859.9170 | ve+ |
| M309T861 | 309.1182 | 860.5800 | ve+ |
| M507T861 | 507.2105 | 861.4530 | ve+ |
| M266T862 | 266.1237 | 861.6060 | ve+ |
| M508T862 | 508.2151 | 861.7170 | ve+ |
| M506T862 | 506.2084 | 861.8680 | ve+ |
| M238T862 | 238.0383 | 862.2390 | ve+ |
| M85T863 | 85.0289 | 862.5940 | ve+ |
| M188T863 | 188.0915 | 863.2840 | ve+ |
| M343T863 | 343.1218 | 863.3540 | ve+ |
| M132T864 | 132.1135 | 864.1000 | ve+ |
| M115T864 | 115.0870 | 864.3340 | ve+ |
| M133T864 | 133.1169 | 864.4960 | ve+ |
| M546T867 | 546.2024 | 867.3990 | ve+ |
| M482T868 | 482.1981 | 868.4860 | ve+ |
| M634T869 | 634.2185 | 868.5800 | ve+ |
| M657T876 | 657.2241 | 875.5100 | ve+ |
| M714T879 | 714.2434 | 878.9660 | ve+ |
| M366T887 | 366.1401 | 887.0720 | ve+ |
| M855T888 | 855.3174 | 887.7140 | ve+ |
| M854T888 | 854.3117 | 887.7600 | ve+ |
| M876T888 | 876.2959 | 887.9420 | ve+ |
| M871T888 | 871.3418 | 887.9520 | ve+ |
| M514T888 | 514.2000 | 888.5000 | ve+ |
| M92T889 | 92.0711 | 888.6110 | ve+ |
| M513T889 | 513.2002 | 888.7020 | ve+ |
| M512T889 | 512.1958 | 888.9830 | ve+ |
| M224T893 | 224.1125 | 893.1550 | ve+ |
| M303T894 | 303.1548 | 893.7980 | ve+ |
| M658T896 | 658.2531 | 895.6840 | ve+ |
| M160T896 | 160.0970 | 896.0260 | ve+ |
| M206T897 | 206.1019 | 897.1180 | ve+ |
| M246T902 | 246.0944 | 901.8680 | ve+ |
| M527T902 | 527.1379 | 901.9450 | ve+ |
| M264T904 | 264.0659 | 904.4070 | ve+ |
| M262T905 | 262.0686 | 904.7520 | ve+ |
| M241T910 | 241.1064 | 909.9400 | ve+ |
| M204T911 | 204.1532 | 910.5750 | ve+ |
| M203T911 | 203.1498 | 910.9280 | ve+ |
| M125T915 | 124.9229 | 915.3360 | ve+ |
| M190T921 | 190.1631 | 920.9670 | ve+ |
| M205T924 | 205.1545 | 924.1840 | ve+ |
| M175T925 | 175.1442 | 924.8720 | ve+ |
| M199T930 | 199.0845 | 929.7260 | ve+ |
| M201T930 | 201.0825 | 929.8670 | ve+ |
| M259T930 | 258.8995 | 930.3460 | ve+ |
| M107T930 | 106.9510 | 930.4230 | ve+ |
| M245T933 | 245.0536 | 932.8560 | ve+ |
| M218T938 | 218.0423 | 937.8590 | ve+ |
| M471T938 | 471.2191 | 937.8880 | ve+ |
| M202T940 | 202.0683 | 940.1000 | ve+ |
| M309T949 | 309.1654 | 949.2660 | ve+ |
| M310T950 | 310.1693 | 949.6310 | ve+ |
| M200T950 | 200.0401 | 950.4500 | ve+ |
| M331T951 | 331.1470 | 951.2350 | ve+ |
| M156T952 | 156.0769 | 951.6480 | ve+ |
| M130T958 | 130.0864 | 957.7200 | ve+ |
| M191T958 | 191.0764 | 957.7260 | ve+ |
| M148T958 | 148.1162 | 957.7300 | ve+ |
| M147T958 | 147.1129 | 957.7320 | ve+ |
| M169T958 | 169.0943 | 957.7420 | ve+ |
| M84T958 | 84.0812 | 957.7780 | ve+ |
| M116T961 | 116.0709 | 960.8580 | ve+ |
| M175T980 | 175.1191 | 980.1960 | ve+ |
