# Supplementary material for: Adaptation of the infant gut microbiome during the complementary feeding transition
Source: PLoS One. 2022 Jul 14;17(7):e0270213. doi: 10.1371/journal.pone.0270213 (PMC9282554; doi:10.1371/journal.pone.0270213)
Supplement: S1 Protocol — (DOCX) [file pone.0270213.s005.docx]

Study Protocol – Feasibility Study

Good Bacteria for Healthy Babies – Complementary Feeding to Nourish the Microbiome that Supports Immune Health “Nourish to Flourish”

BACKGROUND

Weaning is a period of marked physiological change. The introduction of solid foods and the changes in milk consumption are accompanied by significant gastrointestinal tract (GI), immune and developmental adaptations. It is especially in this period of weaning that there are opportunities for the development of complementary food and beverage (F&B) products that aid in the maintenance of optimal health. One key area is the development of the immune system with a focus on the role of GI microbiota.

Recent discoveries have highlighted how the infant immune system co-evolves with GI microbiota in a mutualistic relationship, a crucial event that impacts on the host’s immune system throughout life (1-3), and how aberrant GI microbiota during infancy is associated with disease conditions later in life. Comparative metagenomic studies have underscored the dominant role played by diet in shaping the composition and function of GI microbiota (4, 5). Growth is the most commonly used measure of infant health in research and clinical practice. However, growth as such is a crude measurement. High resolution mMolecular data are needed to understand the mechanisms that define differences in growth rates and subsequent health outcomes. Metabolomics and proteomics provide a functional readout of and insight into the metabolic state of an individual, respectively. Metabolic, enzymatic and protein transporter profiles are influenced by genetics, environment, diet, and - importantly - GI microbiota. Hence, metabolomics and proteomics provide key tools required to understand the interplay between infant feeding, growth, GI microbiota and metabolic health as well as a means to monitor health trajectories non-invasively. In this project, both metabolomic and proteomic technologies will help determine the efficacy of bespoke first-foods targeting specific GI bacterial communities and a means to model the complex relationships that define immune and metabolic health over time.

In this study every infant will be sampled and analysed individually before and after intervention, i.e. being assessed as a case/control pair. This “personal” case/control pair-wise analysis will account for differences in infant feeding (e.g. maternal diet, duration of breastfeeding) and can be interpreted as a multiplexed n-of-1 trial (6).

Preparation for Clinical Pilot (Feasibility Study)

To determine the experimental complementary feed for the clinical pilot study we will bioinformatically exploit existing data and knowledge on immune protection-beneficial bacteria that are present in the infant GI microbiome around weaning and known/suggested to be beneficial for the evolving infant GI and systemic immune system. We will then interrogate the (NZ) food space and identify the best whole foods and food components to feed these beneficial bacteria. This systems biology and ecosystem approach differs fundamentally from classical “test feeding” studies

We will select and focus on infant GI bacteria that are known/suggested to (i) promote healthy immunity in exclusively breast-fed infants and remain post-weaning (7); or (ii) colonise the infant GI microbiome around or after weaning and facilitate healthy immune development. The genomes of these selected infant GI microbes should be available and sufficiently annotated. Suggested candidates for (i) are: *Bifidobacterium longum* subspecies *infantis* (*B. infantis*) (8-10) [Guillaume 2009] and *Bifidobacterium animalis lactis* (*B. lactis*) (11-14). Possible candidates for (ii) can be retrieved from completed infant GI microbiome studies that span the breast feeding, weaning and solid food period along the first year of life (7, 15). From the microbiome angle, we will consult the available genomic, bioinformatic and microbiological information to identify whole foods and food components that support the growth of these beneficial infant GI bacteria. This investigation will extend beyond and complement the established research and knowledge on *Bifidobacteria* specifically consuming human milk oligosaccharides (16).

Correspondingly, from the feeding angle, we will bioinformatically process genomic and biochemical information available in the food space to identify and source key whole foods and food components from NZ’s food chains. We will leverage our domestic food composition and food-health databases (17) and select whole foods and food components that support the growth of the selected beneficial infant GI bacteria that are “generally accepted as safe” (GRAS). Although being an experimental diet applied in an exploratory study, the design of the ECF will ensure that both whole foods and food components will be acceptable to both the consuming babies and the purchasing parents.

STUDY OBJECTIVES

Primary objectives of the clinical pilot feasibility study are:

1. To determine whether infants will consume the target food(s) in a dose estimated to alter immune function via modification of the microbiome and associated metabolite changes.
2. To determine the feasibility of the outcome measurements (immune, microbiome, hot/microbe metabolome), assess recruitment potential, and identify high prevalence adverse events.
3. To determine the number of infants that would be required for an RCT of a complementary feeding intervention for improved immune protection from infection.
4. Which outcome variable(s) are both relevant and feasibly to demonstrate the chain of effects of whole food(s) or food component(s) on immune health benefits?

**Research Approach**

Our research will address the knowledge gaps that currently limit the demonstration of beneficial modulation of protection against infection in infants by NZ F&B complementary feeding products, by means of positively influencing the microbiome. This will be achieved by embracing human individuality and complexity, rather than eliminating it using the traditional reductionist approaches typically employed in this sphere of science. The programme is organised into three work packages:

1. Develop and employ a systems biology approach to utilise public data of bacteria with supporting evidence for beneficial modulation of the evolving infant GI and systemic immune system and;
2. Interrogate NZ whole foods and food components to identify lead candidates to feed these beneficial bacteria identified in (i); and ensure commercial feasibility of these candidates;
3. Conduct a pilot clinical trial, with two experimental (prebiotic) complementary feeding diets, plus a probiotic control, informed by (i) and (ii).

Our research strategy will leverage the systems biology-generated data. This differs fundamentally from classical “test feeding” studies. The envisaged full clinical trial planned as part of Tranche II of HVN, will help de-risk NZ F&B investments in examining the efficacy of complementary infant feeding products.

# **STUDY METHODS/DESIGN**

Ethical Considerations

Ethical approval will be obtained from The Northern B Health and Disability Ethics Committee (HDEC) of the Ministry of Health, New Zealand (HDEC reference number TBC).

Trial Design

Nourish to Flourish is a non-randomised clinical pilot study with an active control involving 40 infants (and mothers) which will examine the feasibility of conducting a randomised controlled clinical trial (RCT) to support immune protection against infection. This study will be conducted in Auckland, New Zealand. The study outline is shown in **Figure 1**.

Participant characteristics

Eligible study participants for the feasibility study are 40 healthy infants 2 to 3 months of age with the expectation that they will be introduced to their first complimentary foods around 6 months of age, and not before 4 months of age, as per the Ministry of Health Food and Nutrition Guidelines (Ref).

Infants will be ineligible for enrolment if they: were born <32 weeks’ gestation; have a developmental disability (i.e., autism, intellectual disability); an illness likely to influence their nutritional status (e.g., a chronic illness known to cause malabsorption, digestive or metabolic disorders); who have a doctor-diagnosed cow’s milk allergy; have health conditions that affect feeding; are undergoing treatment with antibiotics; are receiving a supplement with a pre- and/or probiotic; or whose parents written or spoken English comprehension is likely to make participation difficult for them. The decision to participate and written, informed consent will be obtained by the research team from parents/guardians on the child’s behalf. Infants who were born premature or small for gestational age will also be excluded as they may have special dietary requirements.

Infants will be recruited from urban central and greater areas of Auckland. Recruitment strategies include electronic and print advertising through The University of Auckland, community outreach campaigns including coffee groups, antenatal groups, local newspapers, and appropriate social media platforms.

**Ethical Considerations**

Participation in the study is entirely voluntary. All caregivers (parent or legal guardian) of the participants will be provided with written informed consent. The Informed Consent Form will be signed and dated by the caregiver before they enter the study or undergo the any intervention related to the study. The Investigator or their nominee will explain the details of the trial and provide a Participant Information Sheet, ensuring that the caregiver has had sufficient time to consider their infant participating or not. The Investigator will answer any questions that the caregiver has concerning study participation. The investigator or their nominee shall emphasise to them that consent regarding study participation may be withdrawn at any time without penalty or affecting the quality or quantity of their future medical care, or loss of benefits to which the participant is otherwise entitled.

One copy of the consent will be kept by the caregiver, and one will be kept by the Investigator. The trial will not be initiated before the protocol, informed consent forms and participant information sheets have received approval / favourable opinion from the NZ Health and Disability Ethics Committee (HDEC).

The process for obtaining participant informed consent will be in accordance with the REC guidance, and Good Clinical Practice (GCP) and any other regulatory requirements that might be introduced. The investigator or their nominee and the participant or other legally authorised representative shall both manually sign and date the Informed Consent Form before the person can participate in the study.

The decision regarding participation in the study is entirely voluntary. The investigator or their nominee shall emphasize to them that consent regarding study participation may be withdrawn at any time without penalty or affecting the quality or quantity of their future medical care, or loss of benefits to which the participant is otherwise entitled. No study specific intervention will be done before informed consent has been obtained.

If the Informed Consent Form is amended during the study, the investigator shall follow all applicable regulatory requirements pertaining to approval of the amended Informed Consent Form by HDEC and use of the amended form (including for ongoing participants).

**Study Intervention**

Approximately 40 infants aged 2 to 3 months will be recruited for a 6-month study with the intervention starting at around 6 months of age – The NZ Ministry of Health and World Health Organisation recommended age for introducing complementary foods (ref).

The study will be conducted in two stages to enable completion within the tight timeframe. Thirty (30) of the recruited infants will be receive a dietary intervention based on a prebiotic food (candidate food) and 10 infants will receive a probiotic control known to show relevant immune benefit(s) and accepted as an ingredient in infant nutrition. These 10 infants will serve as a positive control to ensure that our study design and methods in principle can detect probiotic-related immune benefit. Envisaged probiotic controls are: *Bifidobacterium longum subspecies infantis (B. infantis)* (ref) and *Bifidobacterium animalis lactis (B. lactis)* (ref).

Researchers will supply the dietary intervention at no cost to the participant. The food will be given to the infants daily in appropriate amounts according to their age and stage of weaning. The amount of food given to the infant will not displace other foods of important nutrient composition in the infant’s diet. The infant will be introduced to the food with their first complementary foods and the food will be consumed daily for the 6-month period of the intervention.

Parents and guardians will be requested to have their infant consume the supplied candidate food or probiotic control each day for a total of 6 months (i.e. from when the child turns 6 months old until they turn 1 year of age). Adherence will be measured using information from a monthly questionnaire with parents or guardians on the average amount of candidate food consumed per day in the previous month.

Outcomes

Parents/guardians will complete daily records of illness through reporting the incidence, severity, and duration of the 9 signs of upper and lower respiratory tract and GI infections. This will be completed throughout the course of the study. Specific consent will be obtained to access medical records for infection-related GP visits, medication prescription and use, and hospitalisation events from 6 to 12 months of age. The infectious nature of the illness will be established by microbiological data, and/or the use of validated questionnaires.

Data Collection

Data collection will be conducted in a clinical setting for baseline, when the child is 4-6 months (CF1), 9 months of age (CF3), and 12 months of age (CF6). Additionally, brief monthly questionnaires administered over the telephone or as a face-to-face interview will collect information on the dietary intervention adherence and recent health history.

**Questionnaires** (baseline, CF1, CF3, CF6)

From 4 to 6 months, brief monthly questionnaires will determine breastfeeding, formula feeding, and whether/what solids have been introduced. Brief exit interviews will determine participant burden and suggestions to improve engagement with the intervention.

**Anthropometry** (baseline, CF1, CF3, CF6):

Standardised anthropometric measurements will include weight and length. Measurements will be performed using calibrated age-appropriate instruments, including electronic scales and measurement mat with the children wearing light or no clothing. Measurement accuracy will be monitored by two parameters, the degree of refinement (recorded to 0.5 cm or 0.1 kg) and the degree of tolerance (measurement agreement between 1 cm and 0.1 kg). Three serial measurements from each infant will be calculated. Prior to the measurement of weight, infants will be required to have a nappy change if soiled.

**Diet** (CF1, CF3, CF6):

Adherence to the food targets will be determined by daily retrospective questionnaires, product counts, and photographs of food offered and waste. Dietary intake will be measured at CF1, CF3, and CF6 using 3-day weighed food records. Breast milk remains an important component of the diet during the age period in question, and has important impacts on both the GI microbiota and immune function. Breast milk volume will be measured. Infant formula can be captured directly by the diet record.

**Blood** (CF1 and completion):

Specific consent will be sought to collect two blood samples (CF1 and CF6). The blood sample will be collected by a trained phlebotomist and will be used to assess the following:

- The protective antibody response in plasma following the oral rotavirus which is administered in the standard immunisation schedule at 6 week, 3 months, and 5 months of age. (Isolauri 1995)
- The protective antibody response in plasma following the intramuscular pneumococcal vaccination which is administered at 6 weeks, 3 months, 5 months, and 15 months of age. (Bunout 2004, Perez, 2010)

A plasma sample will be obtained for global metabolite profiling and total antibody titres in plasma will be assessed at 6, 9 and 12 months as a non-specific measure of protective antibody responses.

Peripheral blood mononuclear cells will be isolated to perform B cell receptor (BCR) repertoire analysis. The total antibody repertoire encoded by B cells is continuously shaped by exogenous as well as endogenous factors, such as vaccines [Galson 2014] and the GI microbiota [Wesemann 2015], both key variables in the study. The high-throughput analysis of the BCR repertoire, by means of next-generation deep sequencing of the immunoglobulin heavy chain complementary determining region (CDR) [Perez 2010], will provide the necessary depth of analysis to detect potential changes in the clonal diversity of individual antibody repertoires while changes to the microbiota are being made via the proposed nutritional intervention [Boyd 2016, Georgiou 2014].

**Urine** (baseline, CF1, CF3, and CF6)

Urine samples will be taken at collected at 6, 7.5, 9 and 12 months for global metabolite profiling. Clean catch urine samples are the recommended method for urine collection. If not available, other non-invasive methods such as the use of urine collection pads should be used (NICE Guidelines). Parents/caregivers will be provided with all necessary equipment for the successful collection and storage of urine samples. Collection pads for obtaining urine specimens from young children, such as the Euricol® Newcastle Urine Collector (UK). Sample collection will be as follows: parents/caregivers must ensure that the participating child’s genital area is clean and dry (using un-medicated products). The collection pad is applied to the area where urine is voided, i.e. in an infant, the pad can be kept in place with a disposable nappy fitted inside out (so that there is no potential for the nappy to absorb the urine). The pad should be checked every 10 minutes to see if it is wet. If no urine is collected after 30 minutes then a new pad should be applied. Following successful collection on the pad, a syringe should be used to draw urine up out of the pad. Samples should be transported to the clinic within a short time frame, or can be stored in a refrigerator and processed appropriately.

**Stools** (baseline, CF1, CF3, and CF6)

Faecal samples will be collected at 6, 7.5, 9 and 12 months of age according to a pre-defined protocol. Faecal samples will be used to assess the extent to which the complementary feeding has modulated the infant GI microbiota and to quantify vaccine-specific antibody responses as a non-specific measure of protective antibody responses. Parents/caregivers will be provided with all necessary equipment for the successful collection and storage of stool samples from their infants nappy e.g. gloves, sterile sample container with scoop for the stool sample, zip lock bag, polystyrene box, freezer gel pack, and a rubbish bag. Parents/caregivers will collect samples from the nappy after the participating infant has passed a bowel motion. Scoops present in the sample container will transfer approximately 1 tsp of the stool sample into the sterile tube. Once the lid is secure, the sterile tubes will then be placed in the zip lock bag together with the frozen gel pack and placed in the polystyrene box provided. Samples can be kept at room temperature in the polystyrene box and brought into the pre-arranged clinic appointment within 24 hours.

**Saliva** (baseline, CF1, CF3, and CF6)

Saliva samples will be collected to quantify vaccine-specific antibody responses at 6, 9 and 12 months of age as a non-specific measure of protective antibody responses. Samples will be collected according to a pre-defined protocol. Saliva will be collected using a Salivette. The cotton wool swab is removed from the salivette and placed in the infant’s mouth until saturated (1 to 3 mins). This is achieved through rolling the swab inside the child’s cheek. The swab is replaced in the salivette and capped. Once prepared, samples will be stored at -80°C until analysis.

**Breast Milk** (baseline, CF1, CF3, and CF6)

Breast milk samples will be collected (where possible) from breast feeding mothers at baseline, 6, 6.7, 9, and 12 months of age according to a pre-defined protocol. Samples will be used for vaccine-specific titre quantification. Using a breast pump or via hand express, 12 ml of breast milk will be collected. This will be transferred into a 15 ml Falcon tube provided. Collection should take place within 24 hours of the pre-arranged clinic visit. Documentation of the time of day the sample is collected will also be recorded. Samples collected at home will be transported in a cooler with a freezer pack to the clinic. Samples will be stored in the -80°C freezer. If immediate storage in the -80°C freezer is not possible, samples should not be stored in a regular fridge for longer than 4 hours after the time of collection before they are transferred to the -80°C freezer in the boxes provided.

**Multi-omics data generation and systems-level analysis**

The plasma and urine samples will be analysed using gas chromatography-mass-spectrometry (GC-MS) [Chan 2011] and liquid chromatography-mass spectrometry (LC-MS) methods [Fraser 2013a, Fraser 2012]. This global metabolite profiling methodology using a MS based approach monitors low molecular weight (<2000) polar and non-polar metabolites present in biological samples and generates data useful for characterising phenotypes, or determining levels of differentially expressed metabolites by treatments. Identification of significant metabolites will be performed by a combination of searching in-house and public mass spectral and metabolic pathway databases (HMDB, Metlin, MassBank, KEGG) to assist in component identification and interpretation of the function of the metabolites of interest, or if no library match is found, by performing more advanced MSn experiments to elucidate structural information [Fraser 2013b, Wishart 2013]. The team will have access to the most comprehensive Metabolomics Platform in NZ and Australia, i.e. at AgR.

Microbial function will be assessed by shotgun sequencing of metagenomic DNA [Young 2016] using the Illumina HiSeq platform in conjunction with microbial profiling by pyrotag sequencing of bacterial 16S rRNA gene amplicons. Microbial profiling of faecal samples (6, 7.5, 9 and 12 months of age) will be performed first and the data used to select at least two time points for the metagenomic analysis. Sequencing data will be analysed using bioinformatics tools such as Qiime [Caporaso 2010], MG-RAST [Meyer 2008], and IMG [Markowitz 2012]. While increasing the number of specific members of the microbiota is not considered in itself by EFSA a beneficial physiological effect [ESFA 2010)], CFDA has a pre-approved claim based on regulating GIT flora. Better understanding of the microbiota functionality is primordial to understand how the host and the microbiota interact in term of a food and health claim.

Randomisation

N/A

Blinding

Neither the participant nor researcher will be blinded.

Sample Size:

This feasibility study will determine the power for a subsequent RCT there no sample size calculations are required. The proposed sample size of 30 infants who will receive the feeding intervention and 10 infants will receive the active control (probiotic) for a period of 6 months. This sample target is based on an achievable recruitment rate of participants within a 12 month time frame and allows for follow up for 6 months.

In addition to classical case/control analyses, every infant will not only be sampled but also analysed before and after the prebiotic feeding, i.e. being assessed as a case/control pair, in addition to classical data analysis. This “personal” case/control pairwise analysis will account for differences in infant feeding before the actual intervention (e.g. duration of breast feeding, infant formula differences) and can be interpreted as a multiplexed n-of-1 trial.

The feasibility aspect of this study will assess compliance and rate of loss to follow up. This data will be used to inform a subsequent RCT. In previous studies and prevalence studies that we have conducted in 12 to 24 month old children the recruitment rate has been approximately 2 participants per week.

Statistical methods

A multiplexed n-of-1 clinical design trial design in which every infant is sampled before and after the intervention, and followed up. Thereby constituting its own case-control pair.

Data Management

Each participant will be assigned a trial identity code which will be allocated at enrolment to allow for link-anonymisation of data. The documents and database will also use infant date of birth. All personal data will be stored on an encrypted drive with links to personal information only being available to the study co-ordination team. Paper forms and hard copies will be stored in locked filing cabinets in secure sites. Data will be consolidated by each site using the REDCap (Research Electronic Data Capture) software (RW.ERROR - Unable to find reference:1572). This is a secure web application which is password protected and not accessible by third parties. REDCap will be managed at the University of Auckland. The investigator will also keep a screening log with details of all participants screened for the trial with their name, date of birth, NHI number, and trial number. The investigators will permit monitoring, audits, review, and regulatory inspections, providing direct access to source data/documents (where appropriate).

All measures and entered data are double-checked against source documents. Trained personnel will conduct the study according to standard operating procedures and protocols. All incidents or deviations from the protocol will be documented. A major violation is defined as one that may impact subject safety, affect integrity of the study data and/or subject’s willingness to participate in the study. This is a serious discrepancy resulting from error, fraud or misconduct e.g. failure to obtain informed consent or a breach in randomisation procedures. Should a major violation occur HDEC will be notified. Adverse events will be reviewed by an internal clinician; should any events be deemed as concerning for the health of the child they will make a decision as to whether the child’s GP needs notification (if consented to do so) or in more extreme cases, will withdraw the child from the study and make recommendations for clinical care. Should caregivers (parent or legal guardians) not wish to provide GP details, the clinician will contact them directly in situations deemed necessary in his medical opinion. All essential documents, including source documents will be retained for a minimum period of 10 years following the end of the study.

Data Analysis

Integrated analysis of the multi-omics data from multiple platforms will be performed to visualise and interpret changes according to biological pathways and networks. This will be performed using software tools such as Ingenuity Pathway Analysis (IPA), mixOmics [Le Cao 2009], and metaCyc [Caspi 2014]. In additional to classical case/control analyses, every infant will not only be sampled but also analysed before and after the “prebiotic” feeding, i.e. being assessed as a case/control pair, in addition to classical data analysis. This “personal” case/control pairwise analysis will account for differences in infant feeding before the actual intervention (e.g. duration of breast feeding, infant formula differences) and can be interpreted as a multiplexed n-of-1 trial [24].

Clinical Pilot Study Objectives and Milestones

1. To determine whether infants will consume the target food(s) in a dose estimated to be sufficient to impact protection against infection *via* modification of the microbiome and associated metabolite changes.
2. Milestone 1: Amount of the candidate food(s) free-living infants are offered and consumed over the complementary feeding period (6 to 12 months of age).
3. Milestone 2: Best method(s) to collect data on adherence to consuming the candidate foods (number attempted, number completed, quality).
4. To determine the feasibility of the outcome measurements (infection, immune, microbiome, host/microbe metabolome), assess recruitment potential, and identify high prevalence adverse events.
5. Milestone 1: Feasibility of measuring protection against infection using daily parental report of infection (number attempted, number completed, quality).
6. Milestone 2: Feasibility of measuring immune response to the rotavirus and pneumococcal vaccines in venepuncture blood and saliva as measures of immune function (% of children who followed the immunisation schedule, % of parents who agree to blood test, % successful blood draws in young children, data fit to purpose).
7. Milestone 3: Feasibility of using medical records as a measure of immune response
   (% of parents who agree to data access, completeness of data access, data fit to purpose).
8. Milestone 4: Feasibility and logistics of collecting 4 faecal samples between 6 and 12 months of age (number collected).
9. Milestone 5: Recruitment rate per month and extent and type of advertising required.
10. Milestone 6: Prevalence and nature of parent-reported adverse events, and identification of gross changes in growth rate.
11. To determine an appropriate sample size for the proposed full clinical trial.
    1. Milestone 1: Sample size required for the primary objective of the proposed clinical trial – improved immune protection from infection as a result of consuming the candidate food(s).
    2. Milestone 2: Sample size required for the secondary objective of the proposed clinical trial – altered microbiota as a result of consuming the candidate food(s).

Research Plan (2019 – 2024)

The present proposal aims at delivering the scientific insight and development concept to enable design and execution of a follow-up full clinical trial, informed by the pilot trial described in the present submission. The full-scale clinical study will test whether and how immune protection benefits can be conferred to infants around the weaning period by “targeted prebiotic feeding”. In contrast to the pilot trial (conducted at the Liggins Clinical Research Unit) this full trial is envisaged to be set up and performed at the “Starship Children's Health” Hospital, Auckland.

The full CT is conceived to have three arms: (i) one group of infants will receive a prebiotic feed as informed by our ECF developments (Tranche I); (ii) the second group will receive a control complementary feeding (“standard weaning food”) as recommended and accepted in today’s infant nutrition practice; (iii) the third (smaller) group of infants will be given a probiotic control, known to show relevant immune-protection benefit(s) and accepted as infant nutrition ingredient, as a positive control (like in in the pilot trial.)

We plan to extend the omics-based molecular phenotyping from microbiomics, metabonomics, BCR sequencing, and immune cell biology, to proteomics and beyond. This comprehensive molecular phenotype will be correlated with classical clinical readouts. As tested in the pilot CT, we will analyse the full CT data by both classical group comparisons and longitudinally, with every child being analysed individually, before and after the prebiotic complementary feeding. This latter analysis is expected to deliver microbial, immune and health trajectories across the weaning period.

**References**

1. L Drayton D, Liao S, Mounzer R, Ruddle N. Lymphoid organ development: From ontogeny to neogenesis. ; 2006.

2. Eberl G, Lochner M. The development of intestinal lymphoid tissues at the interface of self and microbiota. Mucosal Immunology. 2009 Nov;2(6):478-85.

3. Mazmanian SK, Round JL. The gut microbiota shapes intestinal immune responses during health and disease. Nature Reviews Immunology. 2009 May;9(5):313-23.

4. David LA, Maurice CF, Carmody RN, Gootenberg DB, Button JE, Wolfe BE, et al. Diet rapidly and reproducibly alters the human gut microbiome. Nature. 2014;505(7484):559-63.

5. Muegge BD, Kuczynski J, Kinghts D, Clemente JC, González A, Fontana L, et al. Diet Drives Convergence in Gut Microbiome Functions Across Mammalian Phylogeny and Within Humans. Science. 2011;332(6032):970-4.

6. Lillie EO, Patay B, Diamant J, Issell B, Topol EJ, Schork NJ. The n-of-1 clinical trial: the ultimate strategy for individualizing medicine? Personalized Medicine. 2011 Mar 1,;8(2):161-73.

7. Amarri S, Benatti F, Callegari ML, Shahkhalili Y, Chauffard F, Rochat F, et al. Changes of Gut Microbiota and Immune Markers During the Complementary Feeding Period in Healthy Breast-fed Infants. Journal of Pediatric Gastroenterology and Nutrition. 2006 May;42(5):488–495.

8. Hoy-Schulz YE, Jannat K, Roberts T, Zaidi SH, Unicomb L, Luby S, et al. Safety and acceptability of Lactobacillus reuteri DSM 17938 and Bifidobacterium longum subspecies infantis 35624 in Bangladeshi infants: a phase I randomized clinical trial. BMC complementary and alternative medicine. 2016;16:44.

9. You J, Yaqoob P. Evidence of immunomodulatory effects of a novel probiotic, Bifidobacterium longum bv. infantis CCUG 52486. FEMS Immunol Med Microbiol. 2012 /12/01;66(3):353-62.

10. Chaplin AV, Efimov BA, Smeianov VV, Kafarskaia LI, Pikina AP, Shkoporov AN. Intraspecies Genomic Diversity and Long-Term Persistence of Bifidobacterium longum. PloS one. 2015;10(8):e0135658.

11. Zhu D, Sun Y, Huo G, Yang L, Liu F, Li A, et al. Complete genome sequence of Bifidobacterium animalis subsp. lactis KLDS 2.0603, a probiotic strain with digestive tract resistance and adhesion to the intestinal epithelial cells. Journal of Biotechnology. 2016 February 20,;220(Supplement C):49-50.

12. Meng H, Ba Z, Lee Y, Peng J, Lin J, A. Fleming J, et al. Consumption of Bifidobacterium animalis subsp. lactis BB-12 in yogurt reduced expression of TLR-2 on peripheral blood-derived monocytes and pro-inflammatory cytokine secretion in young adults. ; 2015.

13. Meng H, Lee Y, Ba Z, Peng J, Lin J, Boyer AS, et al. Consumption of Bifidobacterium animalis subsp. lactis BB‐12 impacts upper respiratory tract infection and the function of NK and T cells in healthy adults. Molecular Nutrition & Food Research. 2016 May;60(5):1161-71.

14. Sheikhi A, Shakerian M, Giti H, Baghaeifar M, Jafarzadeh A, Ghaed V, et al. Probiotic Yogurt Culture Bifidobacterium Animalis Subsp. Lactis BB-12 and Lactobacillus Acidophilus LA-5 Modulate the Cytokine Secretion by Peripheral Blood Mononuclear Cells from Patients with Ulcerative Colitis. Drug Research. 2016;66(6):300-5.

15. Bäckhed F, Roswall J, Peng Y, Feng Q, Jia H, Kovatcheva-Datchary P, et al. Dynamics and Stabilization of the Human Gut Microbiome during the First Year of Life. Cell Host & Microbe. 2015 May 13,;17(5):690-703.

16. Xu G, Davis JC, Goonatilleke E, Smilowitz JT, German JB, Lebrilla CB. Absolute quantitation of human milk oligosaccharides reveals phenotypic variations during lactation. J Nutr. 2017;147(1):117-24.

17. Sivakumaran S, Huffman L, Sivakumaran S. The Concise New Zealand Food Composition Tables, 12th edition 2016. 2016.

**Figure 1: Study Timeline**

|  | **Year 1** | | | | | | **Year 2** | | | | | | | | | | | | | | **Year 3** | | | |
| --- | --- | --- | --- | --- | --- | --- | --- | --- | --- | --- | --- | --- | --- | --- | --- | --- | --- | --- | --- | --- | --- | --- | --- | --- |
| **Month** | **Sep-17** | **Oct-17** | | **Nov-17** | | **Dec-17** | **Jan-18** | **Feb-18** | **Mar-18** | **Apr-18** | **May-18** | **Jun-18** | **Jul-18** | **Aug-18** | **Sep-18** | **Oct-18** | | **Nov-18** | | **Dec-18** | **Jan-19** | **Feb-19** | **Mar-19** | **Apr-19** |
| **1^st^ group**  (8 infants) |  | | | | | |  | B (8) |  | | CF1(8) | | | CF3(8) | | | | CF6(8) | |  | | | |  |
| **2^nd^ group**  (8 infants) |  | | | | | |  | | B (8) |  | | CF1(8) | | | CF3(8) | | | | | CF6(8) |  | | |  |
| **3^rd^ group**  (8 infants) |  | | | | | |  | | | B (8) |  | | CF1(8) | | | CF3(8) | | | | | CF6(8) |  | |  |
| **4^th^ group**  (8 infants) |  | | | | | |  | | | | B (8) |  | | CF1(8) | | | | CF3(8) | | | | CF6(8) |  |  |
| **5^th^ group**  (8 infants) |  | | | | | |  | | | | | B (8) |  | | CF1(8) | | | | | CF3(8) | | | CF6(8) |  |
| **Number per month** |  | | | | | |  | **8** | **8** | **8** | **16** | **16** | **8** | **16** | **16** | | **8** | | **16** | **16** | **8** | **8** | **8** |  |
|  |  | | | | | | Recruitment | | | | | |  |  |  | |  | | Sample Analyses | | | | | Data Analyses |
| Key time points |  | |  | | Ethics submission |  |  |  |  |  | First infants CF1 |  |  |  | Last infants CF1 | |  | |  | Last infants CF3 |  |  | Last infants CF6 |  |

B: Baseline enrolment (2- 3 months of age)

CF1: Complementary Feeding Intervention Begins

Food ingredient based on literature search (kumara)

Positive control (prebiotic)

CF3: Complementary Feeding Data Collection

CF6: Complementary Feeding Data Collection

**Table 1: Study Schedule**

|  | **Visit** | **Baseline (B)** | **Complementary Feeding 1 (CF1)**  **(4 – 6 months)** | **Complementary Feeding 3 (CF3)**  **(9 months)** | **Complementary Feeding 6 (CF6)**  **(12 months)** |
| --- | --- | --- | --- | --- | --- |
| **QUESTIONNAIRE** | Q: Mother Profile | x |  |  |  |
|  | Q: Mother Health | x |  |  |  |
|  | Q: Mother Nutrition | x |  |  |  |
|  | Q: Father Health | x |  |  |  |
|  | Q: Family Demographics | x |  |  |  |
|  | Q: Family Exit Questionnaire |  |  |  | x |
|  | Q: Child Health | x | x | x | x |
|  | Q: Child Medications | x | x | x | x |
|  | Q: Child Nutrition and Diet (3 DWFR) | x | x | x | x |
|  | Q: Child GP/Hospital Visits | x | x | x | x |
|  | Q: Child Intervention Adherence |  | x | x | x |
|  | Q: Child Respiratory Infections | x | x | x | x |
| **MEASURES** | M: Child Length | x | x | x | X |
|  | M: Child Weight | x | x | x | x |
| **SAMPLE** | S: Mother Breast Milk | x | x | x | x |
|  | S: Child Venous Blood* |  | x |  | x |
|  | S: Child Saliva Swab | x | x | x | x |
|  | S: Child Urine | x | x | x | x |
|  | S: Child Stool | x | x | x | x |

***** Opt in/out

**Table 2: Biological Sample Collection Plan**

***** Opt in/out

| **Visit** | **Member** | **Type** | **Collected** | **Aliquot Type** | **Aliquot # stored at -80°C** | **Aliquot # Liquid Nitrogen** |
| --- | --- | --- | --- | --- | --- | --- |
| **Baseline** | Mother | Breast Milk | 15 ml falcon tube | Whole breast milk |  |  |
|  |  |  | Storage container |  | 1 |  |
|  | Child | Stool | Nappy | Stool |  |  |
|  |  | Urine | Collection pad | Whole Urine |  |  |
|  |  | Saliva |  |  |  |  |
| **Complementary Feeding 1 (CF1)**  **(4 – 6 months)** | Mother | Breast Milk | 15 ml falcon tube | Whole breast milk |  |  |
|  |  |  | Storage container |  | 1 |  |
|  | Child | Stool | Nappy | Stool |  |  |
|  |  | Venous Blood *(2 – 5 ml) | 2 ml tube | Serum |  |  |
|  |  |  | 2 ml Leucosep tube | Plasma (1 ml) | 1 - 3 |  |
|  |  |  | 2 ml Leucosep tube | RNA |  |  |
|  |  |  | Cryovial | PBMC |  | 1 |
|  |  | Urine | Collection pad | Whole Urine |  |  |
|  |  | Saliva |  |  |  |  |
| **Complementary Feeding 3 (CF3)**  **(9 months)** | Mother | Breast Milk | 15 ml falcon tube | Whole breast milk |  |  |
|  |  |  | Storage container |  | 1 |  |
|  | Child | Stool | Nappy | Stool |  |  |
|  |  | Urine | Collection pad | Whole Urine |  |  |
|  |  | Saliva |  |  |  |  |
| **Complementary Feeding 6 (CF6)**  **(12 months)** | Mother | Breast Milk | 15 ml falcon tube | Whole breast milk |  |  |
|  |  |  | Storage container |  | 1 |  |
|  | Child | Stool | Nappy | Stool |  |  |
|  |  | Venous Blood *(2 – 5 ml) | 2 ml tube | Serum |  |  |
|  |  |  | 2 ml Leucosep tube | Plasma (1 ml) | 1 - 3 |  |
|  |  |  | 2 ml Leucosep tube | RNA |  |  |
|  |  |  | Cryovial | PBMC |  | 1 |
|  |  | Urine | Collection pad | Whole Urine |  |  |
|  |  | Saliva |  |  |  |  |
| **Total samples per family** | | | |  |  |  |
